# Metabolic characterization and pathway analysis of berberine protects against prostate cancer

**DOI:** 10.18632/oncotarget.17531

**Published:** 2017-04-28

**Authors:** Xianna Li, Aihua Zhang, Hui Sun, Zhidong Liu, Tianlei Zhang, Shi Qiu, Liang Liu, Xijun Wang

**Affiliations:** ^1^ Sino-America Chinmedomics Technology Collaboration Center, National TCM Key Laboratory of Serum Pharmacochemistry, Chinmedomics Research Center of TCM State Administration, Metabolomics Laboratory, Department of Pharmaceutical Analysis, Heilongjiang University of Chinese Medicine, Harbin, China; ^2^ State Key Laboratory of Quality Research in Chinese Medicine, Macau University of Science and Technology, Taipa, Macau

**Keywords:** prostate cancer, metabolomics, pathway analysis, metabolome, UPLC-Q/TOF-MS

## Abstract

Recent explosion of biological data brings a great challenge for the traditional methods. With increasing scale of large data sets, much advanced tools are required for the depth interpretation problems. As a rapid-developing technology, metabolomics can provide a useful method to discover the pathogenesis of diseases. This study was explored the dynamic changes of metabolic profiling in cells model and Balb/C nude-mouse model of prostate cancer, to clarify the therapeutic mechanism of berberine, as a case study. Here, we report the findings of comprehensive metabolomic investigation of berberine on prostate cancer by high-throughput ultra performance liquid chromatography-mass spectrometry coupled with pattern recognition methods and network pathway analysis. A total of 30 metabolite biomarkers in blood and 14 metabolites in prostate cancer cell were found from large-scale biological data sets (serum and cell metabolome), respectively. We have constructed a comprehensive metabolic characterization network of berberine to protect against prostate cancer. Furthermore, the results showed that berberine could provide satisfactory effects on prostate cancer via regulating the perturbed pathway. Overall, these findings illustrated the power of the ultra performance liquid chromatography-mass spectrometry with the pattern recognition analysis for large-scale biological data sets may be promising to yield a valuable tool that insight into the drug action mechanisms and drug discovery as well as help guide testable predictions.

## INTRODUCTION

Recent explosion of biological data brings a great challenge for the traditional methods. With increasing scale of large data sets, much advanced tools are required for the depth interpretation problems. As a rapid-developing technology, metabolomics can provide a useful method to discover the pathogenesis of diseases [1–3]. Metabolomics aims to profile the wide range of metabolites which is present in biological samples [4–6]. Recently, ultraperformance liquid chromatography-mass spectrometry (UPLC-MS) has been used to support metabolomics applications to facilitate the identification of complex mixtures of analytes from large-scale biological data sets [7]. Metabolomics has been regarded as a novel method by which to explore the changes in both endogenous metabolites and drug action effect. Nowadays, it is widely application in drug discovery, clinical diagnosis, nutrition, food science and any other fields of human health [8–10].

Prostate cancer is one of the most common malignant tumors affecting elderly men in the worldwide, with an incidence rate of 0.03% [11–14]. And the incidence rate and mortality rate of prostate cancer were increasing rapidly [15, 16]. Research indicated that the occurrence probability of prostate cancer is significantly increases after the age of 40 years, with the increasing occurrence rate it takes patients too much emotional and physical burden [17]. And the risk factors of prostate cancer occurrence are including high-sugar and high-calorie diet, smoking, alcohol consumption, sedentary, insomnia, androgens level, hereditary factors and age factors. Prostate cancer has no obvious symptoms at early stage, thus it usually diagnosis in advanced stage. The common clinical symptoms including acraturesis, dysuresia, less semen, hemospermia, short ejaculation time, frequent micturition, urgent urination, incontinentia urinae, uremia, asynodia, *etc*. Various typical biomarkers have emerged for earlier diagnosis, treatment and monitoring of prostate cancer, such as androgen receptor (AR), serum prostate-specific antigen (PSA), prostate-specific membrane antigen, prostatic acid phosphatase, prostate-Specific G-Protein, prostate cancer antigen 3, transmembrane protease serines to ERG, phosphatase and tensin homolog deleted on chromosome ten (PTEN), Prostate stem cell antigen, vascular endothelial growth factor-C, early prostate cancer antigen 2, *etc*. In clinical treatment of prostate cancer, through combined typical biomarkers earlier diagnosis, surgery therapy, radiation therapy, immunization therapy, androgen deprivation therapy the survival rate of prostate cancer will increase. Nowadays, most of studies were only focused on researching the specific signaling pathways, target genes or regulatory elements of prostate cancer, but lack of in-depth research the pathophysiological mechanism of prostate cancer [18–22]. In this context, more and more scholars were focus on to seeking new alternative medicines with low toxicity and less side-effect for preventing and treating prostate cancer. The latest research showed that natural products had become the main chemotherapeutic drugs for cancer treatment, and more and more natural products had been approved as anticancer agents [23]*.* Berberine is an isoquinoline plant alkaloid, it was widely present in different traditional Chinese medicines and Ayurvedic medicine, such as *Phellodendron Chinensis* Schneid*, Phellodendron Amurense* Rupr., *Berberis Soulieana* Schneid., *Coptis Chinensis* Franch and so on [24–26]. Modern pharmacological research has proved that berberine has various pharmacological activities, including antimicrobial, anti-inflammation, anti-diarrhoea, anti-depressant, anti-ulcerous, anti-diabetes, anti-hypertension, glucose-lowering, cholesterol-lowering, anti leukemic, anti-tumor, enzyme-inhibiting and immune regulation [27–30]. It has been widely utilized in the treatment of arrhythmia, heart-failure, dysentery, diabetes, cancer and many other diseases [20–36]. Research studies have recently demonstrated that berberine had widely anti-tumor activity, its action mechanism containing inhibit the proliferation and induce the apoptosis of cancer cell, inhibit the invasion and migration of cancer cell, anti-cancer angiogenesis, and it is induced by regulate LHRH and VEGF receptors activate, and it has potential pharmacological activity in the treatment of prostate cancer, but the action of mechanism is unclear, thus its development and application on treating prostate cancer has been always limited [37–39].

Therefore, this study was deeply explored the dynamic changes of metabolic profiling in cells model and Balb/C nude-mouse model of prostate cancer, as well as clarify the therapeutic effects and action mechanism of berberine, through using Metabolomics strategy based on UPLC-MS platform. Furthermore, we investigated the biological information of potential metabolite biomarkers via IPA software and clarified the pathogenesis of prostate cancer and explored the underlying target markers of berberine. In the present study, we provided a reliable research data for developing new drugs of anti-prostate cancer. This present research described the discovery and development of berberine on prostate cancer, and then provided a scientific example to highlight the power of the UPLC-MS platform for large-scale biological data sets.

## RESULTS

### Morphological analysis of 22RV1 cell *in vitro*

**Figure 1:**
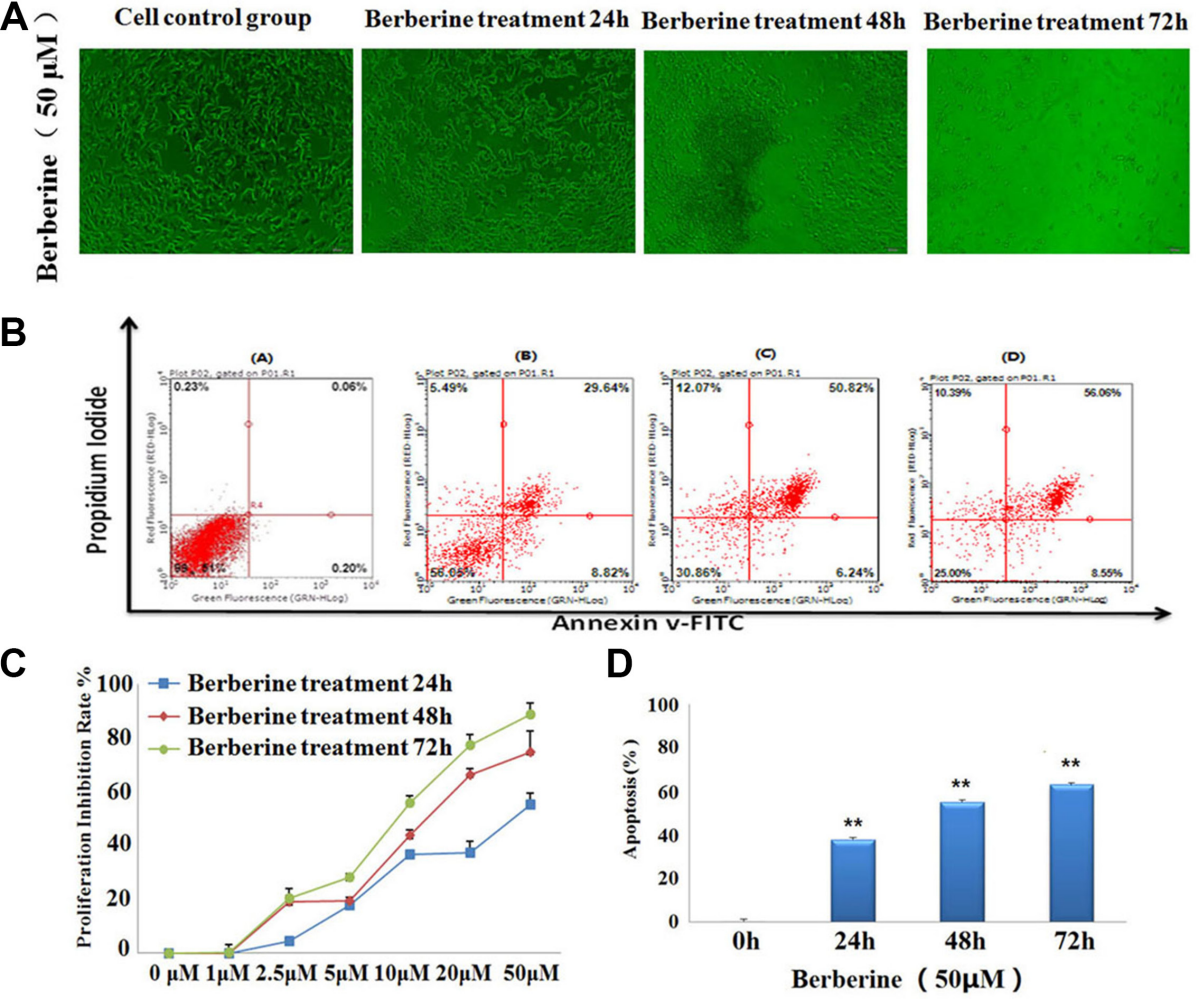
Berberine inhibited the proliferation and induced the apoptosis of 22RV1 human prostate cancer cells (**A**) Berberine caused the cell morphological changes on 22RV1 human prostate cancer cells. (**B**) Different concentrations of berberine inhibited the proliferation of 22RV1 human prostate cancer cells for 24 h, 48 h, 72 h and the cell viability was determined by MTT assay. (**C**) 22RV1 human prostate cancer cells were treated with the concentrations of 50 μM berberine for 24 h, 48 h, 72 h and the cell apoptosis was analyzed with annexin V-FITC/PI staining.

Inverted optical microscope was used to observe the growth status of 22RV1 human prostate cancer cell. Result showed that, the cell morphology was significant changed under the treatment with berberine for 24 h, 48, 72. With the treating time protracting, the cells were growing well in control group, but it was obvious observed that cell growth inhibition and apoptosis increased after treatment with berberine, the cell volume reduced, cell gap grew bigger and cell volume shrink gradually, both of the changing in cell morphology indicated that berberine could inhibited the cell proliferation. The result of 22RV1 human prostate cancer cell morphology was shown in Figure 1A.

### Berberine inhibited the proliferation of 22RV1 prostate cancer cells

In order to investigate the cellular proliferative suppression effect of berberine in 22RV1 human prostate cancer cells, we researched the effect of different concentrations of berberine on prostate cancer cell proliferation using an MTT assay. As shown in Figure 1, the 22RV1 prostate cancer cell treated with less than 10 uM concentration of berberine showed lower inhibitory activity, with the inhibition rate was less than 50%. But the cells treated with 10 μM, 20 μM and 50 μM concentration of berberine exhibited strong inhibitory activity, with the highest inhibition rate value of 88.8%. Result showed that berberine showed strong inhibitory activities for 22RV1 prostate cancer cells proliferation (Supplementary Table 1), with the IC50 value of 13.892 μM and 8.931 μM in 48 h and 72 h, respectively.

### Berberine promote the apoptosis of 22RV1 cell

Apoptosis is a basic form of cell death, and it plays a regulatory role in the process of embryonic development, tissue differentiation and some disease occurrence. In order to detect whether berberine could promote the 22RV1 prostate cancer cell apoptosis, Annexin V-FITC and PI was used for containing to the cells and the apoptosis cells were detected by flow cytometry, the result of cell apoptosis assay was showed in Figure 1B. Result indicated that, the cells in control group was clustered in lower left quadrant of flow cytometry scatter plots, it was means that there was no cell apoptosis in control group. But after 22RV1 prostate cancer cells were treat with berberine for 24 h, 48 h and 72 h at concentrations of 50 μM, the proportion of apoptotic cells increased gradually with the time-dependent. As shown in Figure 1D, the total apoptosis rate was 62.92 ± 2.21% at a concentration of 50 μM after treatment with berberine for 72 h. The results provided a scientific research data that berberine could trigger apoptosis in 22RV1 prostate cancer cells.

### Berberine inhibited the growth of 22RV1 cell

**Figure 2:**
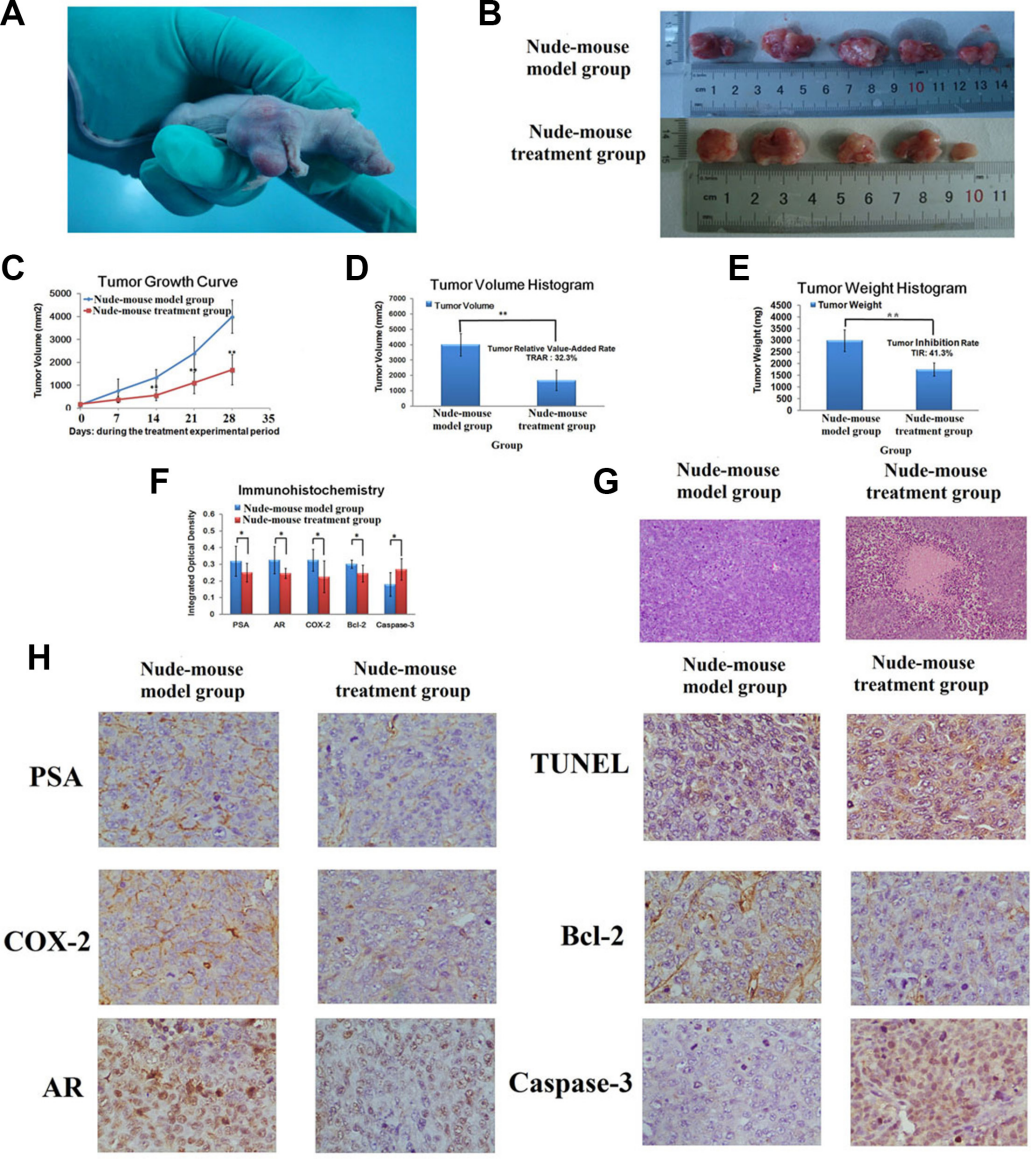
Berberine suppressed 22RV1 prostate cancer cell xenograft growth (**A**) A representative picture of xenograft tumor growth in nude mice subcutaneously inoculated with 22RV1 human prostate cancer cells. (**B**) Representative tumor among model group and treatment group. (**C**) The curve of average tumor volume among model group and treatment group during the treatment experimental period. (**D**) The comparisons of average tumor volume between model group and treatment group for 28 days. (**E**) The comparisons of average tumor weight between model group and treatment group for 28 days. (**F**) The comparisons of IOD value of PSA, AR, COX-2, Bcl-2 and Caspase-3 between model group and treatment group. (**G**) H&E staining of histological evaluation. (Magnification 100×). (**H**) TUNEL analysis and the expression of PSA, AR, COX-2, Bcl-2 and Caspase-3 in IHC analysis. (Magnification 400×). *P < 0.05, **P < 0.01 vs model group.

Result indicated that berberine showed great inhibited effect for 22RV1 human prostate cancer cell - xenograft tumors growth. 15days after injection the 22RV1 human prostate cancer cells, the xenograft tumors were beginning to growth in its inoculation site with the tumor formation rate of 100%, then gradual growth to 21days. Measure the long diameter and short diameter of xenograft tumors, according the formula to calculate the average volume of xenograft tumors, and then began to treat with berberine when both of the volume of xenograft tumors achieved 100–300 mm^2^. During the experimental period, the activities and the body weight of the prostate cancer model nude mice become gradually declined by comparing with nude mice of nude-mouse control group, no nude mice died. During the process of berberine treatment, the average volume of xenograft tumors in nude-mouse treatment group was significant smaller than it in nude-mouse model group (*P* < 0.01). On day 28, the average volume of xenograft tumor in nude-mouse model group and nude-mouse treatment group were 3993.6 ± 725.3 mm^3^ and 1677.8 ± 660.6 mm^3^, respectively (*P* < 0.01). And the relative tumor volume of xenograft tumor in nude-mouse model group and nude-mouse treatment group were 27.8 ± 6.3 mm^3^ and 9.0 ± 2.4 mm^3^, respectively (*P* < 0.05). According the formula to calculate the TRAR of xenograft tumor, result showed that the TRAR of xenograft tumor was 32.4%. At the end of the experiment, the nude mice were killed, then collected and weighted the tumors, the result of tumors weight in nude-mouse model group and nude-mouse treatment group were 2986.8 ± 459.7 mg and 1753.2 ± 279.5 mg, respectively (*P* < 0.01), with TIR was 41.1%. The result of xenograft tumor growth in nude mice subcutaneously, tumor growth curve, tumor volume, relative tumor volume, tumor weight, TRAT and TIR were shown in Figure 2A–2E and Supplementary Table 2. The experimental result indicated that, berberine could inhibit the growth of 22RV1 human prostate cancer cell- xenograft tumors, and it could be regarded as an adjuvant drug in prostate cancer therapy.

### Histopathology, immunohistochemical analysis

Through observation the growth status of the xenograft tumors, result indicated that both of the tumors could move under the skin with the presented spherical or ellipsoid shapes and limited invasion. In nude-mouse model group, the tumors showed a fleshy red surface with white bean dregs tissue or cystic tissue, and some of tumors expression hemorrhage and necrotic. And in treatment group, the tumors exhibited a fleshy pink surface with membrane intact. The result indicated that berberine could effective inhibit tumor growth and invasion ability.

H&E staining result showed that, the cancer cell proliferates quickly with the larger volume and irregularly permutation. But in nude-mouse treatment group, the number and volume of cancer cell was lower and smaller than nude-mouse mode group, the tumor tissue exhibited multiple point or sheet necrosis, karyopyknosis, interstitial fibrous tissue obvious proliferation and lymphocyte infiltration, the H&E staining of histopathology result was shown in Figure 1F. Result reflected that berberine has potential activity to promote 22RV1 tumor cells apoptosis.

The IOD value of COX-2, PAS, AR, Bcl-2 and Caspase-3 expression value of tumor tissues in each groups were determined by IHC analyzing using IPP software. PSA is a specific tumor marker of prostatic cancer [40]. AR plays an important role in the process of prostate cancer growth [41, 42]. COX-2 is an inflammatory mediators, research indicated COX-2 was a potential target for cancer therapy [43]. Bcl-2 and Caspase-3 are the important genes, which were closely related with cell apoptosis [44–46]. After treatment with berberine, the levels of PSA, AR, COX-2, Bcl-2 in tumor tissues were markedly decreased, and the expression of Caspase-3 was increased. The differences of COX-2 and Bcl-2 were evident between the nude-mouse model and treatment group The IOD value of COX-2, PAS, AR, Bcl-2 and Caspase-3 in nude-mouse treatment group and model group were showed in Figure 2F–2H and Supplementary Table 3. Result showed that the effect of berberine treatment on prostate cancer is mainly through inhibiting the inflammatory microenvironment *in vivo*, and then inhibited prostate cancer proliferation and induced prostate cancer apoptosis.

### Berberine promote the apoptosis of 22RV1 prostate cancer cells

The cell apoptosis rate was measured by TUNEL staining, and the result can be determined if berberine had the potential effect of promoting the prostate cancer cell apoptosis (Figure 2H). Each group select 5 high power fields to calculate the percentage rate of apoptotic cells for every 200 cells with the following formula: the cell apoptosis rate = number of positive apoptotic cell/ 200 × 100%. Finally, the cell apoptosis rate of nude-mouse treatment group and model group were (52.6 ± 14.5)% and (21.5 ± 7.5) %, respectively. Obviously, the nude-mouse treatment group had high cell apoptosis rate and the differences were evident between the nude-mouse model group and treatment group (*P* < 0.01 ). It showed good activity of promoting prostate cancer cells apoptosis and then inhibited the growth of prostate cancer tumor.

### Serum metabolomics analysis of berberine on prostate cancer nude mouse

#### Multivariate statistical analysis of metabolome

**Figure 3:**
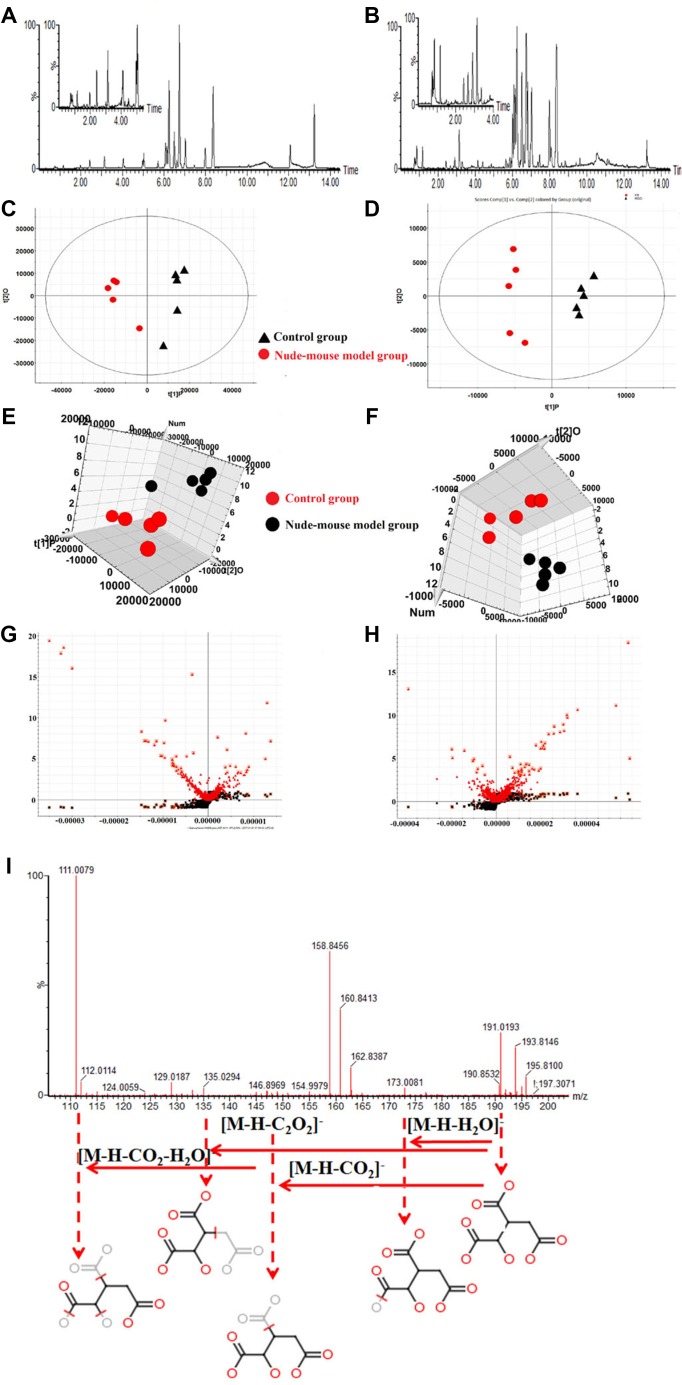
Serum metabolomics analysis (**A**) UPLC-Q/TOF-MS/MS BPI serum chromatograms in positive mode; (**B**) UPLC-Q/TOF-MS/MS BPI serum chromatograms in negative mode; (**C**) PCA score plots for control and nude-mouse model group in positive mode; (**D**) PCA score plots for control and nude-mouse model group in negative mode; (**E**) 3D score plots of OPLS-DA based on serum metabolites discriminating control and nude-mouse model group in positive mode; (**F**) 3D score plots of OPLS-DA based on serum metabolites discriminating control and nude-mouse model group in negative mode; (**G**) Serum metabolite biomarkers in the VIP and S-plot between control and nude-mouse model group in positive mode; (**H**) Serum metabolite biomarkers in the VIP and S-plot between control and nude-mouse model group in negative mode; (**I**) Chemical structure and mass fragment information of isocitric acid in negative mode.

Here we displayed the different metabolic profiles in control group and nude - mouse model group for the first time. The basic peak intensity (BPI) chromatograms of the serum samples in each group were showed in Figure 3A and 3B. We explore the serum biomarkers using Progenesis QI software and EZinfo 2.0 software. Progenesis QI software was used for pre-processing the raw data obtained from UPLC/MS of each group, and then EZinfo 2.0 software was used for further multivariate data analyses, such as unsupervised PCA analysis, supervised PLS-DA analysis and OPLS-DA analysis. Unsupervised cluster and PCA analysis of serum samples provided that there was an obvious biochemical perturbation between the clustering of control group and nude-mouse model group. We identified the potential metabolites between the prostate cancer nude-mouse model and control samples using VIP-plot and S-plot obtained from OPLS analysis coupled with a Student’s *t*-test (*P* < 0.05). In S-plot diagram the farther away metabolite ions from origin represent the higher VIP value of the ions, and the higher VIP value represent the greater contribute to the difference between nude-mouse model group and control group, we selected the ions with VIP value greater than 3 and further confirmed to the potential metabolite biomarkers of prostatic cancer. The PCA, 3D-plot, VIP-plot and S-plot were shown in Figure 3C–3H.

### Metabolite identification

**Figure 4:**
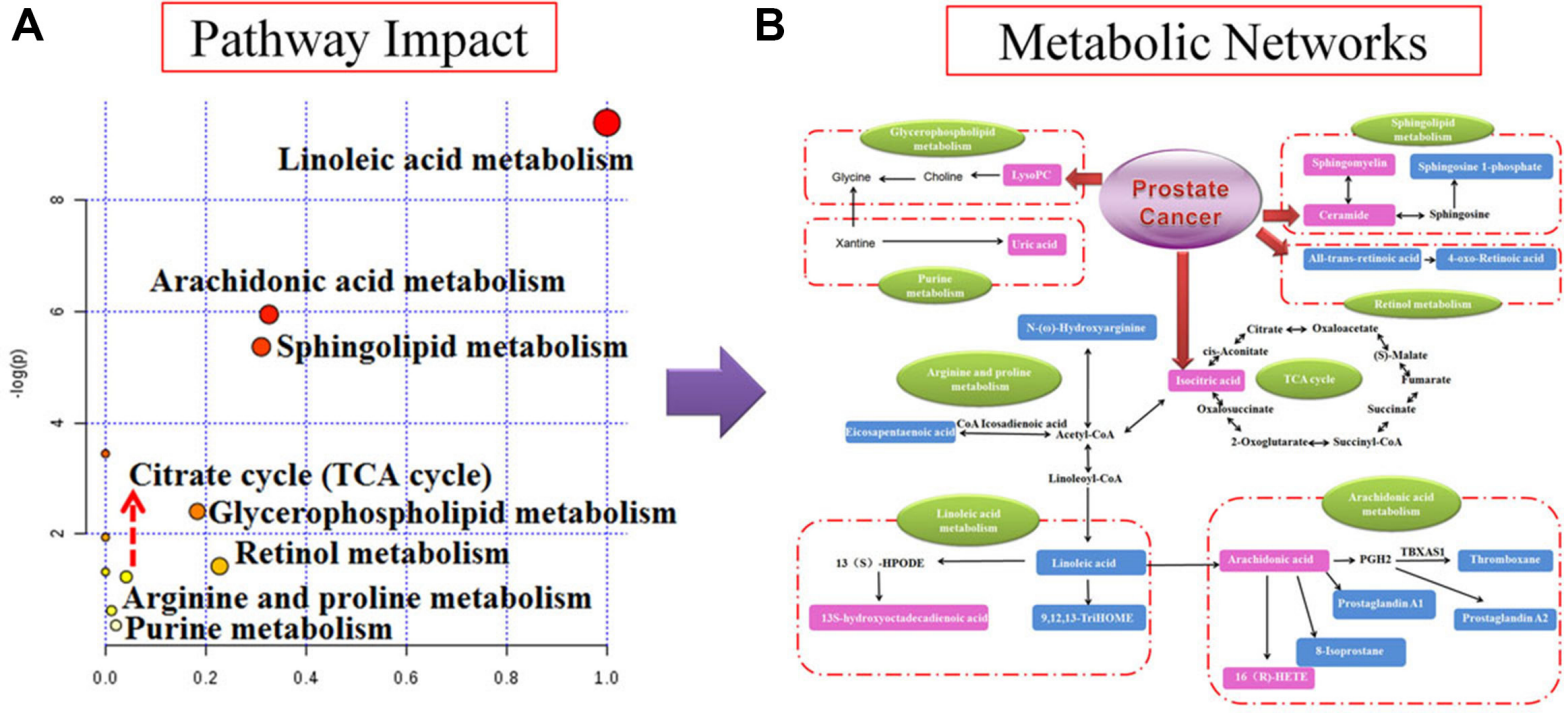
Pathway and metabolic networks analysis (**A**) Pathway analysis with MetaboAnalyst tool; (**B**) Construction of the altered metabolic network associated prostate cancer model based on KEGG pathway database.

UPLC-Q/TOF-MS/MS detection platform was used for collecting the raw data of serum samples, and then identified the potential metabolite biomarkers by the data of retention time, precise MS and MS/MS. Further characterized the structure of metabolite biomarkers and analyses the related metabolic pathways based on reference recorded and on-line database analyzed. According to the analysis method described above, a total of 30 serum metabolite biomarkers (VIP > 3.0, *P* < 0.05) were identified and it was listed in Supplementary Table 4, which were differential levels in control group versus nude - mouse model group. Of these 30 serum metabolite biomarkers, 16 were increased and 14 were decreased by compared with control samples. Some of these serum metabolite biomarkers have been reported to be closely associated with prostate cancer progression, such as arachidonic acid, thromboxane, 16(R)-HETE, prostaglandin A1, prostaglandin A2, eicosapentaenoic acid, cholines, citric acid and uric acid. For example, the identification process was as follows, ESI negative ion mode gave an [M-H] ^-^ ion at m/z 191.0193 and Rt in 0.89 min with high VIP value, Based on the MS and MS/MS data obtained, the molecular formula of the ion was speculated of C_6_H_8_O_7_, the MS/MS screening were observed at m/z 191[M-H]-; 173[M-H-H_2_O]-; 148[M-H-CO_2_]-; 135[M-H-C_2_O_2_]^-^; 111[M-H-CO_2_-H_2_O]-. Finally, it was characterized as isocitric acid through searching the on-ling databases. The mass spectrum and proposed fragmentation pathway of isocitric acid were showed in Figure 3I. MetaboAnalyst platform was used for determining the metabolic pathways associated with potential biomarkers of prostate cancer, and to select the impact value was greater than 1 as the prostate cancer related metabolic pathway. Result showed that 8 metabolism pathways were closely related to the development of prostate cancer, including arachidonic acid metabolism, glycerophospholipid metabolism, linoleic acid metabolism, purine metabolism, sphingolipid metabolism, retinol metabolism, citrate cycle (TCA cycle), arginine and proline metabolism. The result of metabolic pathways analysis was showed in Figure 4A and Supplementary Table 5. And according to analysis the correlation between metabolite biomarkers and metabolic pathways, the metabolic networks of metabolite biomarkers were detailed description in Figure 4B.

### Protective effects of berberine in prostate cancer nude mouse

**Figure 5:**
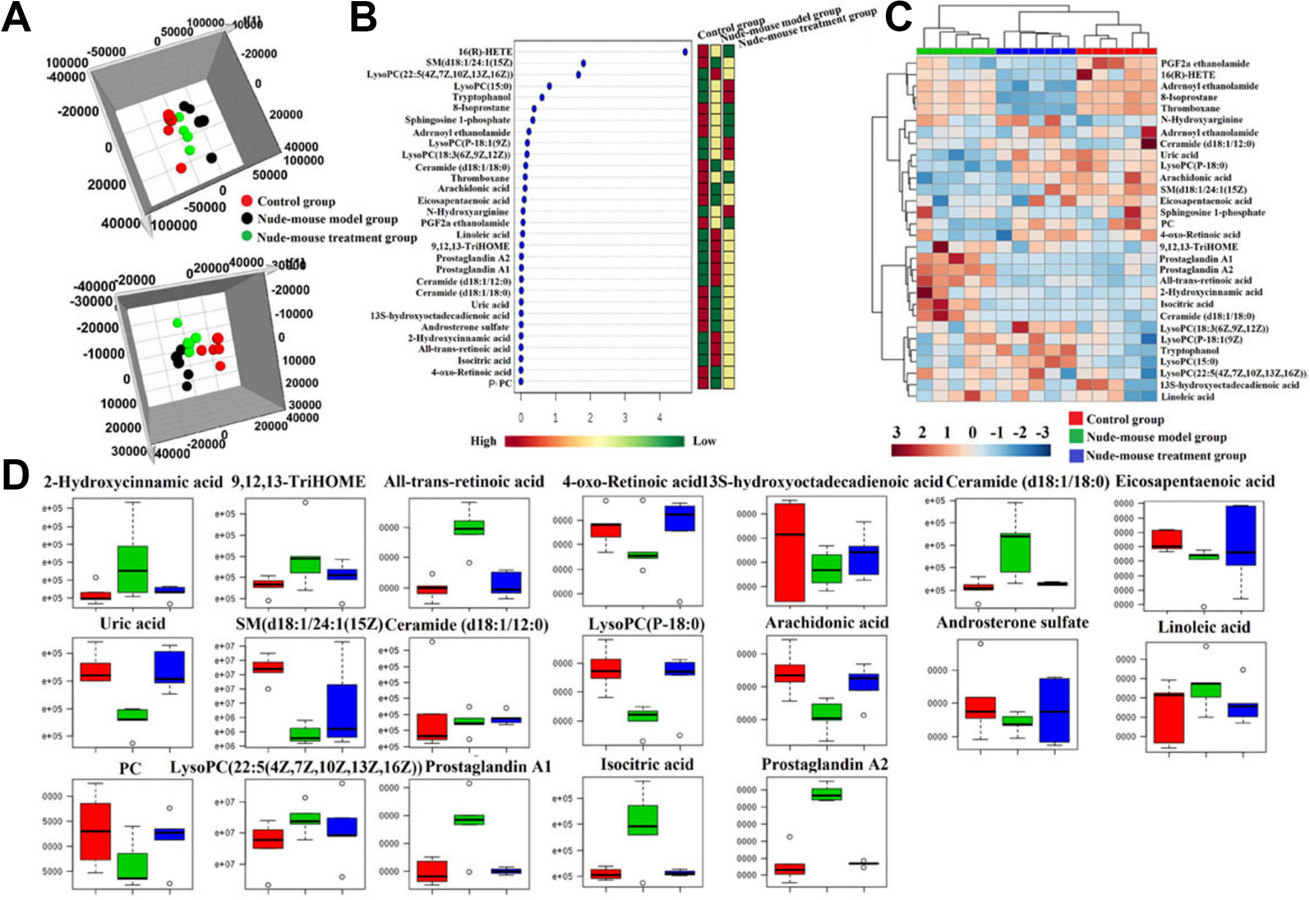
Serum metabolic profiling characterization and multivariate data analysis (**A**) 3D score plots of OPLS-DA based on serum metabolites discriminating control group, nude-mouse model group and nude-mouse treatment group in both positive and negative mode; (**B**) VIP scores of the serum metabolite marker candidates; (**C**) The heatmap visualization for serum samples from the control, model group and treatment group; (**D**) Relative signal intensities of the serum metabolites identified by UPLC-Q/TOF-MS/MS.

For visualizing the difference metabolic profiling among the different groups, PCA analysis and hierarchical clustering analysis was used to analyze the expression levels of metabolic biomarkers in different groups. Result showed that the metabolic profile of nude-mouse treatment group was closer to the control group than the nude-mouse model group, it was indicated that berberine can effectively reverse the abnormal metabolic profile of prostate cancer, the results were showed in Figure 5A. All the raw data were put into MetaboAnalyst system analysis to further discover the effects of berberine against prostate cancer. Hierarchical cluster analysis result was showed that the metabolic profile in treatment group were close to the control group, it was indicated that berbeine had strongly biological activity in treating prostate cancer and the heatmap was showed in Figure 5C. Through analysis the VIP scores of biomarkers in different groups, it was showed that the relative concentration of these metabolites could be reversed after taking the berberine, the VIP scores were showed in Figure 5B. Through analysis the level of identified metabolite biomarkers in the control group, nude-mouse model group and nude-mouse treatment group, berberine can be completely reversed 19 biomarkers to abnormal level and it was showed in Figure 5D., including increased the expression level of 4-oxo-Retinoic acid, 13S-hydroxyoctadecadienoic acid, eicosapentaenoic acid, uric acid, SM(d18:1/24:1(15Z), ceramide (d18:1/12:0), lysoPC(P-18:0), arachidonic acid, androsterone sulfate, PC(22:6(4Z,7Z,10Z,13Z,16Z,19Z)/22:4(7Z,10Z,13Z,16Z)), and decreased the expression level of 2-hydroxycinnamic acid, 9,12,13-TriHOME, all-trans-retinoic acid, ceramide (d18:1/18:0), linoleic acid, lysoPC (22:5(4Z, 7Z,10Z,13Z,16Z)), prostaglandin A1, isocitric acid, prostaglandin A2 in serum of prostate cancer model nude-mouse. Through input the 17 potential biomarkers into MetaboAnalyst platform, the metabolic pathways which was associated with berberine against prostate cancer were determined. Result showed that 7 metabolism pathways were associated with the process of berberine against prostate cancer, including purine metabolism, citrate cycle (TCA cycle), linoleic acid metabolism, arachidonic acid metabolism, retinol metabolism, sphingolipid metabolism and glycerophospholipid metabolism. Result indicated that berberine has obvious biological activity in the treatment of prostate cancer.

### Metabolomic analysis of berberine on prostate cancer cells

#### Multivariate statistical analysis

**Figure 6:**
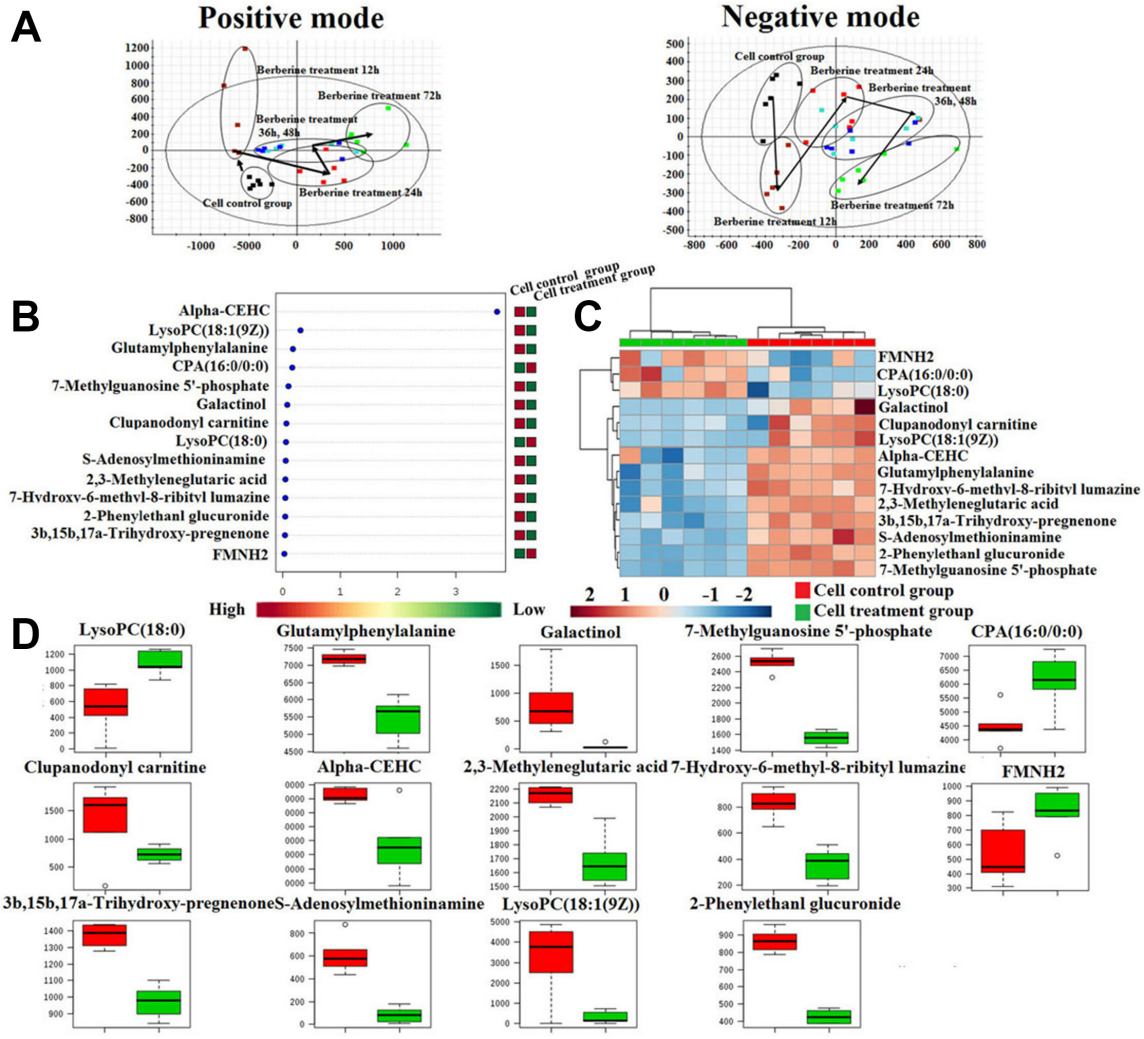
Cell metabolic profiling characterization and multivariate data analysis (**A**) The PCA score plots of 22RV1 prostate cancer cells treatment of berberine at 12 h, 24 h, 36 h, 48 h and 72 h in both positive mode and negative mode; (**B**) VIP scores of the cell metabolite marker candidates; (**C**) The heatmap visualization for 22RV1 prostate cancer cell samples from the cell control group and cell treatment group; (**D**) Relative signal intensities of the cell metabolites identified by UPLC-Q/TOF-MS/MS.

In order to determine the effects of berberine against prostate cancer, we studied the viability of prostate cancer cells that were treated with 10 μM for the following 12 h, 24 h, 36 h, 48 h and 72 h. UPLC-Q/TOF-MS/MS detection platform was used for collecting the raw data of prostate cancer cell samples, and then put the raw data into Progenesis QI software (Waters, Milford, USA) for data pre-processing. EZinfo software (Waters Corp., Milford, USA) was used for further multivariate data analyses, such as PCA, PLS-DA and OPLS-DA. PCA-score plots was shown in Figure 6A, it suggested that prostate cancer cell metabolic profiles were significantly separated and turn to the direction away from model group with the prolongation of treatment time. It is indicated that berberine could effectively reverse the metabolic profile of prostate cancer cells, especially of administration berberine for 72 h. Then put the raw data into MetaboAnalyst on-line database to further discover the effects of berberine against prostate cancer. Hierarchical cluster analysis result was showed that the metabolic profile in cell treatment group were obviously different from cell control group, it was indicated that berberine had clearly therapeutic efficacy, the heatmap were showed in Figure 6C. The screening conditions for potential biomarkers selection were set as VIP value greater than 3 and *P* value less than 0.05 from the OPLS analysis and Student’s *t*-test. And then characterized the structure of potential biomarkers and analyses the related metabolic pathways based on reference recorded and on-line database analyses using precise molecular MS and MS/MS value with mass error less than 5 ppm.

### Metabolite identification and metabolic changes after berberine exposure in cell

Through analysis the level of cell metabolic biomarkers of the cell model group and treatment group for administration berberine of 72 h, totally 14 cell metabolic biomarkers of berberine resistance to prostate cancer cell proliferation were identified, including 3 were increased and 11 were decreased in treatment group by compared with cell mode group. The result of cell metabolic biomarkers was listed in Supplementary Table 6. and the Vip-scores and relative signal intensities of cell metabolic biomarkers was showed in Figure 6B and 6D. Through input the 14 potential biomarkers into MetaboAnalyst platform, the metabolic pathways which were associated with berberine resistance to prostate cancer cell proliferation were determined. Result showed that 3 metabolism pathways were associated with the process of berberine resistance to prostate cancer cell proliferation, such as Phenylalanine metabolism,D-Arginine and D-ornithine metabolism and Tyrosine metabolism. Result indicated berberine could significantly reversed the metabolic profiling of prostate cancer cells, further indicated that berberine has obvious biological activity in treating prostate cancer.

### Potential target prediction of berberine against prostate cancer

**Figure 7:**
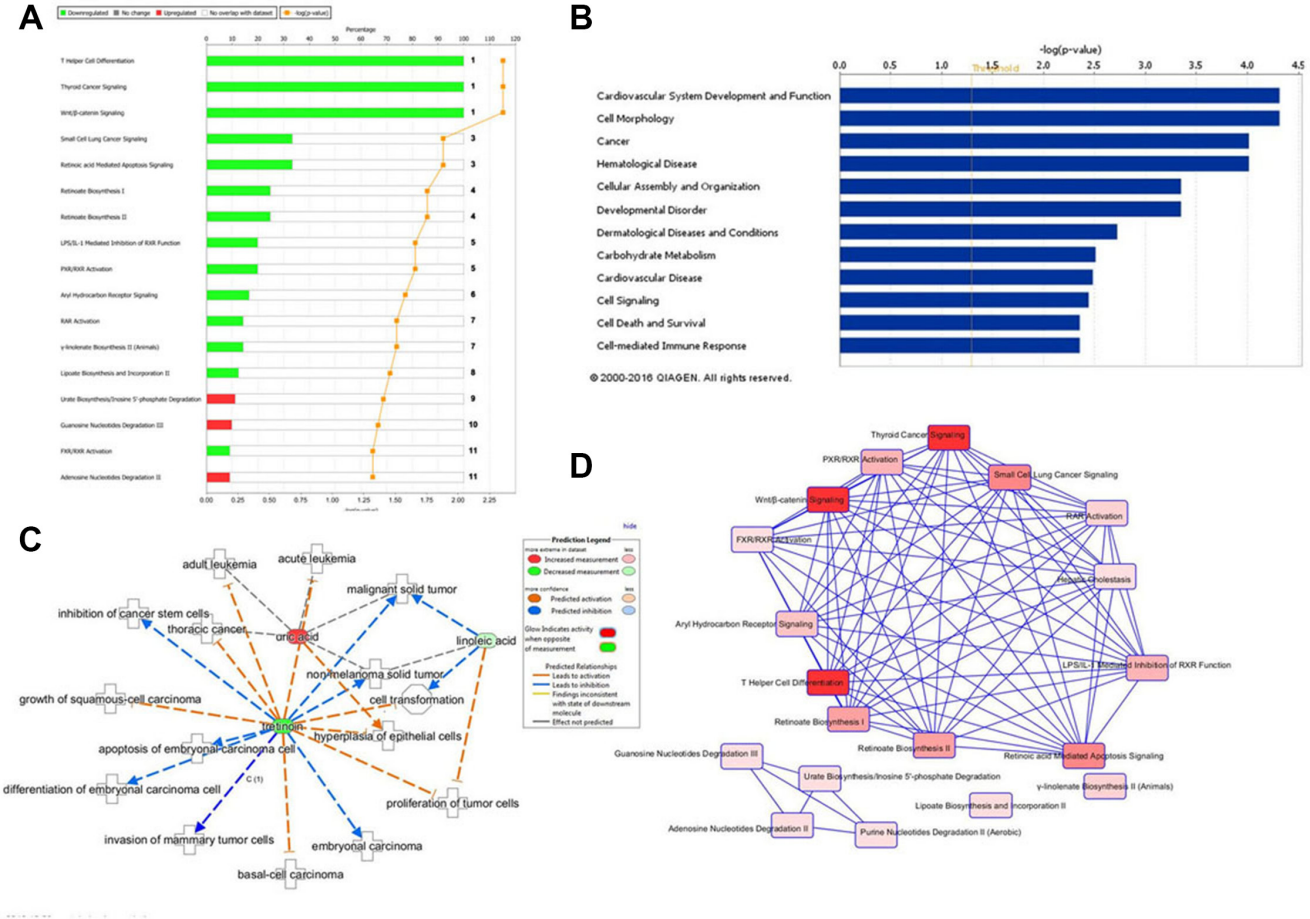
Ingenuity pathways analysis of metabolite biomarkers (**A**) Top canonical pathways identified by IPA that are searched of serum metabolites; (**B**) The biological functions of serum metabolite biomarkers; (**C**) The biologically active functions network of main serum metabolite biomarkers; (**D**) IPA analysis reveals a network of signaling pathways searched by serum metabolite biomarkers.

Ingenuity Pathway Analysis (IPA) online software was used for deep investigated the raw data and comprehensive analysis the functions of potential metabolite biomarkers of berberine against prostate cancer based on prostate cancer nude mouse model and cell model, and then to clarify the action mechanism of berberine against prostate cancer from a dynamic view of whole body metabolism. Compared with the model group of prostate cancer nude mouse, berberine could reverse the abnormal level of 19 metabolite biomarkers, and then imported these biomarkers into the IPA software for pathway and network analysis and predict the potential targets, 14 differentially expressed metabolite biomarkers were mapped, including 13-hydroxyoctadecadienoic acid, 2-furoic acid, 2-hydroxycinnamic acid, PC, 5ʹ-deoxyadenosine, 9,12,13-TriHOME, androsterone sulfate, beta-tyrosine, SM, linoleic acid, prostaglandin A1, prostaglandin A2, All-trans-retinoic acid and uric acid. The top canonical pathways of these biomarkers were T helper-cell differentiation, thyroid cancer signaling, small cell lung cancer signaling, retinoic acid mediated apoptosis signaling and so on, the result was showed in Figure 7A and 7D. To further investigate the relative disease and bio-functions among the altered metabolites and explore the main metabolite biomarkers of berberine against prostate cancer based on prostate cancer nude mouse, result showed that these reversed metabolite biomarkers was most relative with inflammatory response and cancer, and its molecular and cellular functions were associated with cell morphology, cellular development, cellular growth and proliferation, the relative disease and bio-functions result were showed in Figure 7C and 7B. Cell metabolomics analysis result showed that 12 significantly altered metabolite biomarkers were observed in prostate cancer cells treated with berberine. Through input these biomarkers into the IPA software for pathway and network analysis and predict the potential targets, 9 differentially expressed metabolite biomarkers were mapped, including lysoPC(18:1(9Z)), CPA(16:0/0:0), alpha-CEHC, 7-hydroxy-6-methyl-8-ribityl lumazine, 7-methylguanosine-5’-monophosphate, glutamylphenylalanine, L-alpha-lysophosphatidylcholine, stearoyl, FMNH2, s-adenosylmethioninamine. The top canonical pathways of these biomarkers were Spermine biosynthesis and α-tocopherol degradation, the result was showed in Supplementary Figure 1A and 1B. To further investigate the relative disease and bio-functions among the altered metabolites and discover the main metabolite biomarkers of berberine against prostate cancer based on prostate cancer cells, result showed that these reversed metabolite biomarkers were most relative with infectious disease, and its molecular and cellular functions were associated with release of arachidonic acid, lipid metabolism and molecular transport, the relative disease and bio-functions result were showed in Supplementary Figure 1C. Through comprehensive analysis the bioinformatics of serum metabolite biomarkers and cell metabolite biomarkers via IPA software, result indicated that the main metabolite biomarkers of berberine against prostate cancer were uric acid, linoleic acid, tretinoin, 13-hydroxyoctadecadienoic acid, 1-oleoyl lysophosphatidylcholine, S-adenosyl-3-methlthiopropylamine and L-alpha-lysophosphatidylcholine stearoyl.

In order to deep investigate the interactions among the important metabolite biomarkers and further explore the action mechanisms of berberine against prostate cancer, IPA software was used for the bioinformatics analysis of metabolite biomarkers. The biological network was indicated that the important signaling molecules were Vegf, ERK1/2, p38 MAPK and PLA2G7, as well as some enzymes and protein, such as NADPH oxidase, SMAD3 and ODC1, the IPA network was showed in Supplementary Figure 2. The metabolite pathways and the key action target of berberine treatment prostate cancer was analyzed by the IPA software, it demonstrated that berberine displayed an obvious effect in treatment of prostate cancer through reversing the abnormal expression of metabolite biomarkers and adjusting the disturbed metabolic pathways.

## DISCUSSION

In the present study, we used UPLC-Q/TOF-MS for comprehensive analysis the metabolic profiling and metabolites in serum samples and cell samples of berberine against prostate cancer by prostate cancer nude-mouse model and 22RV1 prostate cancer cells model. Result showed that berberine could effective reversed the abnormal metabolism of prostate cancer. After deeply investigated the bioinformatics of reversed serum metabolite biomarkers and cell metabolite biomarkers via IPA software, 7 core metabolite biomarkers were identified, including uric acid, linoleic acid, tretinoin, 13-hydroxyoctadecadienoic acid, 1-oleoyl lysophosphatidylcholine, S-adenosyl-3- methlthiopropylamine and L-alpha-lysophosphatidylcholine stearoyl, these core biomarkers were closely related to the occurrence and development of prostate cancer. Then we mapped the related metabolite pathways of berberine against prostate cancer via ultra-modern software of IPA software and KEGG database, 6 pathways were identified, including linoleic acid metabolism, purine metabolism, retinol metabolism, arginine and proline metabolism, spermine biosynthesis and retinoate biosynthesis. The IPA demonstrated that important biochemical molecules such as VEGF, ERK1/2, p38 MAPK and PLA2G7, NADPH oxidase, SMAD3 and ODC1 were involved in the biological network associated with berberine against prostate cancer.

LysoPC(18:1(9Z)) (1-oleoyllysophosphatidylcholine), lysoPC(18:0) (L-alpha-lysophosphatidylcholine stearoyl), lysoPC(P-18:0), lysoPC(15:0), lysoPC(18:3(6Z,9Z,12Z)), lysoPC(P-18:1(9Z))) were markedly changed in the serum and cell of prostate cancer, these markers were involved in two main metabolic pathways, including glycerophospholipid metabolism and choline metabolism. Research had indicated that abnormal choline metabolism was closely related to the occurrence and development of cancers, phosphatidylcholine could coordinate the integrity of the cell membrane and the abnormal expression of phosphatidylcholine was a potential target in the diagnosis of cancers [47–49]. And the abnormal expression of choline kinase-a, ethanolamine kinase-a, phosphatidylcholine-specific phospholipase C, phosphatidylcholine-specific phospholipase D, glycerophosphocholine phosphodiesterases and any other molecules are the main causes of choline metabolism disturb *in vivo*. Result indicated that the expression of total choline, free choline, phosphocholine and glycerophosphocholine were significantly higher in prostate cancer calls, and abnormal expression of phospholipids was associated with malignant diseases. In the research of prostate cancer, the researchers found that the expression of total choline and its metabolites were abnormal in prostate cancer. In this experiment, we found PC (22:6(4Z,7Z,10Z,13Z,16Z,19Z)/22: 4(7Z,10Z,13Z,16Z) was express abnormally in serum of prostate cancer node-mouse. It was suggested that the occurrence of prostate cancer caused the release of inflammatory factors, and then lead to the damaged of the matrix membrane, and affected the choline metabolism. LysoPC(18:1(9Z))(1-oleoyllysophosphatidylcholine), lysoPC(18:0)(L-alpha-lysophosphatidylcholine stearoyl), SM(d18:0/16:1(9Z)), lysoPC (18:0) and lysoPC(18:1(9Z)) and sphingosine 1-phosphate were the signaling molecules of glycerophospholipid metabolism, the significant changed in these metabolites predict the cell growth disorder caused by the disorder of glycerophospholipid metabolism, and it was a major hallmark of cancer occurred. After treatment with berberine, these abnormal metabolite biomarkers were observed to recover to the normal level. The IPA demonstrated that SMAD3 influenced downstream lysoPC(18:1(9Z)) (1-oleoyl lysophosphatidylcholine) and LysoPC(18:0) (L-alpha- lysophosphatidylcholine stearoyl) and it was showed in Supplementary Figure 2. Previous studies have proven that SMAD3 is a downstream signal transduction molecule of TGF-β signaling pathway, it is a antioncogene and down-expression in some malignant tumors [50]. TGF-β is involved in many basic biological functions including cell migration, cell growth and apoptosis, cell adhesion, cell differentiation, tumor invasion and metastasis [51, 52]. Berberine could effectively regulate SMAD3 and then affected the TGF-β signaling pathway, and then to treated prostate cancer.

Uric acid has antioxidant activity, and it can prevent the progression and reduce the mortality of cancers, such as lung cancer, colorectal cancer, prostate cancer and so on, and serum uric acid is influences the incidence rate and mortality of cancers [53]. Uric acid was involved purine metabolism pathway, it was a product of purine nucleotide and comes from the oxidation of xanthine and hypoxanthine, and eliminated by the intestinal tract and kidney into urine [54]. The high concentrations of uric acid could inhibited lipid peroxidation, and then lead to clear the free oxygen radicals based on xanthine oxidoreductase action. Allantoin was an oxidation product of uric acid, it is the sign of cell oxidative stress. Previous research has shown that oxidative stress was associated with inflammation, and long-term inflammation leads to tumorigenesis. Uric acid could promote cell apoptosis, scavenging oxygen radicals, inhibit lipid peroxidation, and then as an antioxidant to prevent the deterioration of the tumor [55, 56]. The uric acid level changed significantly in the blood of prostate cancer nude-mouse model, the reactivity of oxygen radicals enhanced and lead to tumorigenesis. It suggested that berberine could effectively increased the expression level of uric acid and then decreased the oxidative damage.

S-Adenosylmethioninamine (dcSAM), and N(ω)-hydroxyarginine were involved in arginine and proline metabolism, cysteine and methionine metabolism. DcSAM was a chemical intermediate in the synthesis of polyamine, and it was involved in cell growth and differentiation [57]. Recent research showed that dcSAM was a potential adenovirus-mediated target of antisense molecule in many cancer call lines. In the tumor tissues, the increasing expression level of polyamine could lead to uncontrolled cell proliferation, thus the overexpression of polyamine was closely related to the occurrence of cancer [58]. It showed that the increased expression of polyamine biosynthesis enzymes in prostate cancer cells promoted the increased expression of S-adenosylmethionine. N (ω) -hydroxyarginine is a product of arginine-NO signaling pathway. N (ω) –hydroxyarginine through inhibited the synthesis of arginase, and finally inhibits the cell proliferation. After treatment with berberine, S-Adenosylmethioninamine and N(ω)-hydroxyarginine were gradually observed to recover to the normal level, it was means that berberine could effectively inhibit the cell proliferation and then prevent the development of prostate cancer.

4-oxo-retinoic acid and all-trans-retinoic acid were involved in retinol metabolism. All-trans-retinoic acid is a metabolic intermediate of vitamin A and has various biological activities. Recent review demonstrated that retinol metabolism plays an important role in the process of cancer occurrence and development, it could inhibit cancer cell proliferation, promote cancer cell apoptosis, promote cancer cell differentiation, inhibit chemical induce cancer occurrence and activate the immune system. Retinoic acid is the active substance of retinol regulates gene expression. Reported showed that the retinol, retinoic acid, retinoic ester level decreased significantly in human prostate cancer cells compared with human prostate epithelial cells, suggesting that prostate cancer could induce abnormal expression of tryptophan metabolism [59, 60]. In the present study, we considered that retinol metabolism showed severe disturbance in the progress of prostate cancer. After treatment with berberine, the abnormal expression of 4-oxo-retinoic acid and all-trans-retinoic acid reversed normal state, and it could control abnormal expression of tryptophan metabolism.

Linoleic acid and 13-hydroxyoctadecadienoic acid were involved in linoleic acid metabolism, and linoleic acid was the key substance of arachidonic acid synthesis [61]. In the present research, arachidonic acid, thromboxane, 16(R)-HETE, prostaglandin A1, prostaglandin A2 and 8-isoprostane were involved in arachidonic acid metabolism. Arachidonic acid metabolism was most related with the process of tumor genesis, metastasis and therapy. Arachidonic acid is 5,8,11,14- eicosatetraenoic acid, it is essential fatty acids for human beings, it was the precursor of various bioactive substances and had various physiological functions. Arachidonic acid was present in cell membrane with the form of phospholipids, when the cell membrane was strongly stimulated, it will release from phospholipids and converse into the active metabolite of 16(R)-HETE. Phospholipase A2 and TXA2 were made from arachidonic acid under the catalysis of cyclooxygenase. And arachidonic acid could made of HETEs, leukotrienes and lipoxins under the function of lipoxygenase. Ios-prostane was the predominant metabolic products of arachidonic acid. It is widely known that arachidonic acid was associated with cancer cells proliferation and growth [62]. Furthermore, thromboxane could promote cancer cell proliferation through enhancing the activity of cell mitosiscell mitosis [63]. Thus, because of cell proliferation and apoptosis imbalance, eventually leading to the occurrence of prostate cancer.

To further discover the action mechanism of berberine against prostate cancer, IPA software was used to build a biological network, and then to discover the important signaling molecules and enzymes associated with the relative biomarkers. VEGF, ERK1/2, p38 MAPK, NADPH oxidase, SMAD3 and ODC1 were the important signaling molecules in this biological network. For example, VEGF, ERK1/2, p38 MAPK, NADPH oxidase were the downstream signal molecule of linoleic acid, uric acid, 13-hydroxyoctadecadienoic acid and tretinoin. ERK1/2 influences downstream VEGF and NADPH oxidase, ERK1/2 was closely related to cell proliferation and differentiation and induced cell apoptosis, and further arrested tumor growth. The growth and transfer of cancers depend on formation of new blood vessels. VEGF was one of the important pro-angiogenic factor, it could promote angiogenesis and make cancer cells suffering hypoxia, and further promote cancer metastasis and invasion. VEGF had been detected in multiple tumor tissues. In modern cancer study, VEGF has become a novel targets for cancer therapy. P38 MAPK participates in downstream multi-signaling, such as P38 MAPK influences downstream NADPH oxidase, VEGF and ERK1/2. VEGF could induce metastasis of cancer cells through P38MAPK signaling pathway, thus blocking P38MAPK signaling pathway could inhibits VEGF induced cancer cells metastasis. NADPH oxidase was related with oxidative stress, and the activation of NADPH oxidase was an important symbol of oxidative stress levels increased. Furthermore, NADPH oxidase had been demonstrated to be related with the occurrence and development of cancer [64].

## MATERIALS AND METHODS

### Chemicals and materials

Methanol and acetonitrile are HPLC grade (Merck, Darmstadt, Germany). Distilled water used for prepare all the aqueous solutions and mobile phase, it was obtained from Watson’s Food &Beverage Co. Ltd (Guangzhou, China). Formic acid, Leucine enkephalin was obtained from Sigma-Aldrich (St. Louis, MO, USA). Rabbit anti-COX-2 monoclonal antibody, Rabbit anti-Bcl-2 monoclonal antibody, Rabbit anti-AR monoclonal antibody, Peroxidase-conjugated affini-pure goat anti-mouse IgG (H+L), Mouse anti-PSA monoclonal antibody, Peroxidase- conjugated affini-pure goat anti-rabbit IgG (H+L), 3, 3-diaminobenzidine (DAB) kit, In situ cell death detection kit POD were purchased from the Roche Applied Science (Roche, Germany). Cell culture reagents RPMI1640 and TRIPSIN 0.25% (1×) were purchased from Hyclone (Logan, Utah, USA). Phosphate buffer saline (PBS) and Fetal bovine serum (FBS) was obtained from Gibco Laboratories (Grand Island, USA). Dimethyl sulfoxide (DMSO) was obtained from Amresco Limited- Liability Company (Houston, USA). 22RV1 human prostate cancer cell was purchased from Cobioer Biosciences Company (Nan Jing, China). Standards of berberine were purchased from the Tianjin Chemical Reagent Co. (Tianjin, China).

### Animal handing

Male BALB/c-nude mice (4-6-weeks-old) used in this study were supplied by the Laboraory Animal Center of Slac Laboratory Animal Company Ltd (Shanghai, China). The handing of the BALB/c-nude mice were in according with the standard of Specific Pathogen Free (SPF), and they were housed in an SFP room maintained at a constant temperature (24 ± 2°C) and relative humidity (50 ± 5%) with a 12 h/12 h dark/light cycle. In order to make sure all of the BALB/c-nude mice could adapt to the experiment environment, they were fed with standard laboratory water and food ad libitum for 7 days before the animal experiment started. Fifteen animals were randomly divided into three groups (control group, nude-mouse model group and nude-mouse treatment group) with five animals in each group. The BALB/c-nude mice in nude-mouse model group and nude-mouse treatment group were subcutaneously injected with 0.2 ml 22RV1 cells suspension on the right forelimb at a density of 4.5 × 10^7^/ml of PBS media. And the BALB/c-nude mice in control group were only injected the same volume of PBS solution. When the volume of xenograft tumors was reached 100–300 mm^3^, the experiment was started. Body weights and the longest and shortest diameter were measured one times a week, and then calculated the tumor volume (V), relative tumor volume (RTV) and tumor relative value-added rate (TRAR) according with the following formula: Volume (V) = (length×width2) / 2; relative tumor volume (RTV) = VT/VO (VT: tumor volume measured at time point, VO : tumor volume measured before experiment); tumor relative value-added rate (TRAR) = TRTV/MRTV×100% (TRTV: RTV of treatment group, CRTV: RTV of mode group). The standards of berberine was dissolved in 0.5% carboxymethyl cellulose solvent and ultrasonic for 30 min to prepare the berberine suspension solution (0.001136g•ml-1). Nude-mouse treatment group was treated with berberine suspension solution 0.01136g/kg intragastrically daily for 28 days. And the control group and nude-mouse model group were received the equivalent volume of 0.5% carboxymethyl cellulose solvent in the same way. Before the end of the experiment for 24 hours all the experimental animals were fasted of food but were free to access water. This study was carried out in strict accordance with the recommendations in the Guide for the Care and Use of Laboratory Animals of the Heilongjiang University of Chinese Medicine. The protocol was approved by the Committee on the Ethics of Animal Experiments of the Heilongjiang University of Chinese Medicine (Permit Number: CEAE-HUCM-20150128).

### Cell culture and berberine treatment

#### Morphological analysis of 22RV1 cell and MTT assay

The 22RV1 human prostate cancer cells were cultured in RPMI 1640 solution (containing with 10% FBS and maintained in incubator at 37°C with 5% CO2. 22RV1 human prostate cancer cells were seeded in 6 well plates adding per well 1 mL cell suspension (2 × 10^5^) and routine cultured for 24 hours. After 24 h, the medium was renewed and then treated with berberine at the concentrations of 50 μM for the following 24 h, 48 h and 72 h. Using inverted optical microscope to observe the growth status of 22RV1 human prostate cancer cell. The effect of berberine inhibit of 22RV1 human prostate cancer cells proliferation was measured using the 3-(4, 5-dimethylthiazol-2-yl)-2,5 diphenyltetrazolium bromide (MTT) assay. Working solution was prepared as follows: berberine dissolved in DMSO with the final concentration of 10 mM/mL, and then dilution of the RPMI1640 culture solution with different concentrations of berberine. 22RV1 human prostate cancer cells were seeded in 96 well plates adding per well 1 mL cell suspension (2 × 10^5^) and routine cultured for 24 hours. After 24 h the medium was renewed and then treated with various concentrations of berberine (1 μM, 2.5 μM, 5 μM, 10 μM, 20 μM, 50 μM) for the following 24 h, 48 h and 72 h, control group was added the equal volume of DMSO, each group was set six parallel wells. Subsequently, discard the culture medium, 20 uL MTT solution (5 mg/mL) was added to each well and continue incubated for 4 hours. After incubation for 4 hours, each well was terminated incubate and added 150 uL DMSO. The plates were then shaken with low speed, until both of the crystals were dissolved. Finally measured optical density value (OD) at 570 nm in an enzyme-linked immunosorbent meter. And then calculate the inhibition rate of each concentrations of berberine with the following formula: inhibition rate% = (1-OD treatment group/OD control group) ×100%. Finally calculate the IC50 values were calculated by SPSS 19.0 (*n* = 6).

#### Apoptosis analysis

The effect of berberine promote 22RV1 human prostate cancer cells apoptosis was monitored using the Annexin V fluorescein isothiocyanate (Annexin V-FITC)/propidium iodide (PI) assay. 22RV1 human prostate cancer cells were seeded into 24-well plates at a concentration of 1 × 10^6^ /well in RPMI 1640 solution (containing with 10% FBS and treated with berberine at a concentration of 50 μM for 24 h, 48 h and 72 h, respectively. Then to rinse the 22RV1 prostate cancer cells with 10 ml PBS solution (137 mM NaCl, 2.7 mM KCl, 4.3 mM Na2HPO4-7H2O, 1.4 mM KH2PO4, adjust pH to 7.4) for two times. And make the 22RV1 prostate cancer cells (1 × 10^6^ cells/ml) resuspended in 1× Binding Buffer. 10 μl RAPID™ media binding reagent and 1.25 μl of Annexin V-FITC were added into 0.5 ml cell suspension (5 × 10^5^ cells) and incubated for 30 min in the dark at 18–24°C. Centrifuge at 1000×g for 5 min at room temperature and then remove media. Gently resuspend 22RV1 prostate cancer cells in 0.5 ml cold 1× Binding Buffer and added 10 μl of propidium iodide (PI). At then replaced the 22RV1 prostate cancer cells on ice and away from dark to analyze by flow cytometry immediately. The different quadrant of the flow cytometry scatter plots represent different cells: lower left quadrant represent viable cells, upper left quadrant represent necrotic cells, lower right represent early apoptotic cells and upper right quadrant represent late apoptotic cells. The sum of early apoptosis rate and late apoptosis rate represents the total apoptosis rate.

#### Cell metabolomics study

The 22RV1 human prostate cancer cells were cultured in RPMI 1640 solution (containing with 10% FBS solution and maintained in incubator at 37°C with 5% CO2. 22RV1 human prostate cancer cells were seeded in 48-well plates adding per well 1 mL cell suspension (2 × 10^5^) and routine cultured for 24 hours. After 24 h, the medium was renewed and then treated with berberine at the concentrations of 10 μM for the following 12 h, 24 h, 36 h, 48 h and 72 h. Collecting the prostate cancer cells in each group and then used for cell Metabolomics analysis.

### Biosample collection and preparation

#### Serum samples

The blood was collected from the eyeball on the 28th day of the experiment. And then set the collected blood into 1.5 ml clean centrifuge tube, centrifuge at 3000 rpm for 15 min at 5°C and then removed serum for the further analysis. The protein in the 50 ul serum samples were deposited with 800 µL methanol, collected the layer of methanol of 800 µL and dried with nitrogen gas at room temperature. The residues were redissolved with 200 µL methanol and extracted in an ultrasonic water bath for 1 min. Before the metabolomics analysis, both of the samples were filtered through the filter membrane (pore size: 0.22 µm) and then inject 5 µL for metabolomics analysis. Quality control sample (QC) was prepared as a mixed sample, which was mix by 5 µL serum samples of each collected samples. QC was used in the optimization of the metabolomic analytical method and monitors the acquire data quality.

#### Tumor samples

Both of the experimental mice were sacrificed at 28th day of the experiment, and then collected the tumors immediately. Weight the tumors and calculate the inhibition rate of tumor growth as the following formula: tumor inhibition rate (TIR) = (MW-TW)/MW×100%, MW: the average weight of nude-mouse model group at the end of experiment, TW: weight of treatment group at the end of experiment. Both of the tumors were stored in 10% formalin for histopathology and immunohistochemical analysis ( IHC ).

#### Cell samples

The prostate cancer cell was collected after treatment with berberine of 12 h, 24 h, 36 h, 48 h and 72 h. Further set the collected cells into 5 ml clean centrifuge tube, centrifuge at 1500 rpm for 5 min at 4°C and removed supernatant. Then to rinse the 22RV1 prostate cancer cells with 10 ml PBS solution and centrifuge at 1500 rpm for 5 min at 4°C for two times. Discarded the supernatant and quenched cells with 1 ml cold methanol, and ultrasonic wave disruption the cells in ice bath with 40 kHz ultrasound frequency and 20 W power for 3 min. Then both of the cell samples were centrifuged at 1500 rpm for 5 min at 4°C and filtered through the filter membrane (pore size: 0.22 µm) for cell metabolomics analysis.

### Histopathology, immunohistochemical and TUNEL analysis

Both of the tumors were fixed by formalin for 12 h. Then made tissues into paraffinembedded blocks and stained with hematoxylin and eosin (H&E). The morphology of tumor tissues were observed by light microscopy and images at 400×magnification were acquisition by Motic Medical 6.0 software (Xiamen Motic Software Engineering Co., Ltd). The expression of COX-2, PAS, AR, Bcl-2 in tumor tissues were analyzed by IHC staining. The performed of IHC analysis as follows: primary antibody was used to incubate the paraffin sections at 4°C for 12 h and rinsed with PBS for 3 times, added the secondary antibody (COX-2, PAS, AR, Bcl-2 monoclonal antibody) with temperature of 37°C for 15 min and rinsed with PBS for 3 times, added the DAB chromogen with room temperature for 3 min and rinsed with distilled water, and then hematoxylin used for counterstaining. The integrated optical density (IOD) value of COX-2, PAS, AR and Bcl-2 in nude-mouse model group and nude-mouse treatment group were quantified by Image-Pro Plus (IPP) software. TUNEL analysis was used for observing the apoptosis of tumor tissues in nude-mouse model group and nude-mouse treatment group. The process of TUNEL analysis was performed according to the instructions of In Situ Cell Death Detection kit, POD. Briefly, Using xylene to deparaffinized the tissue sections, and then washed with xylene for two times, rehydrated with ethanol of gradient concentrations (100%, 95%, 90%, 80%, 70%) for 3 min. Pepsin working solution was used for permeabilized cells for 60 min at 37°C, washed with PBS 2 times and each time for 5 min. TUNEL reaction mixture solution including 50 ul enzyme solution (TdT) and 450 ul label solution ( fluorescein-dUTP ). 50 ul TUNEL reaction mixture solution were added to each sections and incubated for 60 min in the dark at 37°C, washed with PBS 3 times and each time for 5 min. 50 ul converter POD (anti-fluorescein antibody-POD) solution were added to each sections and incubated for 30 min at 37°C, washed with PBS 3 times and each time for 5 min. Finally added 50–100 ul DAB chromogen and incubated for 5 min at 15–25°C, washed with PBS solution 3 times and each time for 5 min. The apoptotic cells of tumor tissues were observed by light microscopy, each group select 5 high power fields to calculate the apoptosis rate for every 200 cells with the following formula: The apoptosis rate ( % ) = number of positive apoptotic cell/ 200 × 100%.

### Metabolomics study

#### Serum metabolomics analysis of berberine against prostate cancer

#### UPLC analysis

Waters Acquity^TM^ ultra performance LC system (Waters, Milford, MA, USA) and Masslynx^TM^ (V4.1) control software were used for analying the serum samples. Using ACQUITY UPLC BEH C18 column (2.1 × 100 mm, 1.7 μM, Waters Corporation, Milford, USA) to separating the serum samples with the injection volune of 5 µL, and the colume temperature was 30°C with flowing rate of 0.2 mL/min. Through comprehensive investigation of chromatography parameters, finally the optimum mobile phase was consist of A water with 0.1% formic acid (H20: HCOOH=100:0.1) and B acetonitrile with 0.1% formic acid (CH3CN: HCOOH=100:0.1). The linear elution gradient program was used as follows: 0–1.5 min, 98–86% A; 1.5–2 min, 86–78% A; 2–4 min, 78–40% A; 4–5 min, 40–35% A; 5–8 min, 35–30% A; 8–10 min, 30–2% A.

#### MS analysis

UPLC system was coupled with a Waters Synapt^TM^ G2-Si High Definition TOF Mass system (Waters, Milford, USA) equipped with electrospray ionization (ESI) source in both positive and negative ion modes were used for comprehensive analyzing the serum samples. The optimized analytical MS conditions were set as follows: the capillary voltage in positive mode and negative mode were set at 2.6 kV and 2.4 kV, respectively; sampling cone voltage, 35.0 V; MS source temperature, 110°C; desolvation gas temperature, 350°C; desolvation gas flow, 600 L/h. The full-scan mass ranges were set at *m/z* 100–2300 Da with 0.3 s duration. The collision energy in low energy scans and high energy scans were set at 10–30 eV and 20–40 eV, respectively. Leucine-enkephalin was used for ensuring to obtain the accurate mass during the MS acquisition. The data collection mode was set of centroid and the process of data acquisition was controlled with Masslynx^TM^ (V4.1) software.

### Metabolomics study in 22RV1 human prostate cancer cell

#### UPLC analysis

Waters Acquity^TM^ ultra performance LC system (Waters, Milford, MA, USA) and Masslynx^TM^ (V4.1) control software were used for analying the serum samples. Using ACQUITY UPLC BEH C18 column (2.1 × 100 mm, 1.7 μM, Waters Corporation, Milford, USA) to separate the serum samples with the injection volune of 3 µL, and the colume temperature was 40°C with flowing rate of 0.4 mL/min. Through comprehensive investigation of chromatography parameters, finally the optimum mobile phase was consisted of A water with 0.1% formic acid (H20: HCOOH=100:0.1) and B acetonitrile with 0.1% formic acid (CH_3_CN: HCOOH=100:0.1). The linear elution gradient program was used as follows: 0–1.0 min, 99–99% A; 1.0–2.0 min, 99–60% A; 2.0–5.0 min, 60–25% A; 5.0–8.0 min, 25–1.0% A.

#### MS analysis

UPLC system was coupled with a Waters Synapt^TM^ G2-Si High Definition TOF Mass system (Waters, Milford, USA) equipped with ESI source in both positive and negative ion modes were used for comprehensive analying the serum samples. The optimized analytical MS conditions were set as follows: the capillary voltage in positive mode and negative mode were set at 3.0 kV and 2.8 kV, respectively; the sampling cone voltage in positive mode and negative mode were set at 30 V and 35V, respectively; MS source temperature, 110°C; desolvation gas temperature, 350°C; desolvation gas flow, 600 L/h. The full-scan mass ranges were set at *m/z* 100–2300 Da. The collision energy in low energy scans and high energy scans were set at 10–30 eV and 20–40 eV, respectively. Leucine-enkephalin was used for ensuring to obtain the accurate mass during the MS acquisition. The data collection mode was set of centroid and the process of data acquisition was controlled with Masslynx^TM^ (V4.1) software.

### Multivariate statistical analysis

Both of the LC/MS raw data was preprocessed by Progenesis QI 1.0 software (Nonlinear Dynamics, 2014, version: 1.0) and then using EZinfo 2.0 software (Waters Corp., Milford, USA) for further multivariate data analyses, including principal components analysis (PCA), partial least-squared discriminant analysis (PLS-DA) and orthogonal partial least-squared discriminant analysis (OPLS-DA). Pareto scaling transformation and data normalization were applied to the data processing before multivariate statistical analysis. The contribution rate of potential biomarkers in metabolic profiling could be determined by VIP-plot, which was obtained by OPLS-DA analysis. Using VIP-plot to select VIP value was more than 3 and Student’s *t*-test to select *P*-value was less than 0.05 as the potential metabolite biomarkers of prostate cancer.

### Identification of biomarkers and metabolic pathway

MassLynx^TM^ software was used for pre-charactering the structural formula of the potential biomarkers. UPLC-Q/TOF-MS/MS detection platform was used for collecting the raw data of serum and cell samples, it could give the information of retention time, precise MS, and then obtain the MS/MS data base on multilevel collision energy (10–30V, 20–40V). Then based on the information of exacting mass measurement and precise MS/MS fragments of the potential biomarkers, it could pre-character the accurate mass and structure of potential metabolite biomarkers. Finally, the structure of metabolite biomarkers were determined through retrieved from the online database of ChemSpider, HMDB, KEGG, METLIN or Lipid Maps with mass error was less than 5 mDa. Then import the potential biomarkers into MetaboAnalyst platform (http://www.metaboanalyst.ca/) to analyze the relative metabolic pathways which were associated with prostate cancer.

### Potential target prediction of berberine against prostate cancer

In this study, we selected the potential serum metabolite biomarkers and cell metabolite biomarkers of prostate cancer. And further imported these metabolite biomarkers into the IPA software (Ingenuity, Redwood City, USA) for bioinformatics analysis the interplay among metabolites, genes, enzymes and proteins, and then combined with KEGG database (http://www.genome.jp/kegg/) to comprehensively analyze the relative metabolite pathways. Further discover the key action target of berberine therapy for prostate cancer.

### Statistical analysis

Both of the Metabolomics raw data were analyzed by Progenesis QI 1.0 software (Nonlinear Dynamics, 2014, version: 1.0) and then using EZinfo 2.0 software (Waters Corp., Milford, USA). The mean ± standard deviation ($\bar{x_{}}$ ± s) of volume, weight, relative tumor volume, tumor relative value-added rate and tumor inhibition rate were calculated by SPSS V19.0 data software package. Multivariate statistical analysis and one-way analysis of variance (ANOVA) method were used for charactering and analyzing the expression level of the potential metabolite biomarkers in each groups (VIP > 3 and *P* < 0.05). Statistical analysis was determined by the Student’s *t*-test. The *P* value of less than 0.05 was considered statistically significant, and *P* value of less than 0.01 was considered extremely statistically significant.

## CONCLUSIONS

In this work, we established the blood metabolomics and cell metabolomics based on UPLC-Q/TOF-MS/MS tool, further comprehensively analyzed the therapeutic effect and action mechanism of berberine against prostatic cancer. This research was deeply systematic evaluate the therapeutic effect and discovered the action mechanism of berberine against prostate cancer. It showed that the growths of xenograft tumors were effectively inhibited by berberine in nude mice. After treatment with berberine, the expression of PSA, AR, COX-2 and Bcl-2 were significantly reduced and the expression of Caspase-3 was significantly increased in tumor samples. TUNEL analysis showed that berberine could effectively promote the apoptosis of prostate cancer cells. 30 blood metabolic biomarkers and 12 cell metabolic biomarkers were discovered, respectively. Furthermore, berberine could reverse the abnormal expression of metabolic biomarkers and regulate the abnormal metabolic profile to the normal state. Moreover, 7 key metabolite biomarkers and 7 important biochemical molecules were identified. These biomarkers were closely related to the occurrence and development of prostate cancer. Berberine could treat prostate cancer by regulating the linoleic acid metabolism, purine metabolism, retinol metabolism, arginine and proline metabolism, spermine biosynthesis and retinoate biosynthesis. Our research could provide the data basis and scientific methods in subsequent studies of berberine therapy for prostate cancer.

## SUPPLEMENTARY MATERIALS FIGURES AND TABLES


