# Supplementary material for: Metabolic characterization and pathway analysis of berberine protects against prostate cancer
Source: Oncotarget. 2017 Apr 28;8(39):65022–41. doi: 10.18632/oncotarget.17531 (PMC5630309; doi:10.18632/oncotarget.17531)
Supplement: Supplementary file 1 [file oncotarget-08-65022-s001.pdf]

Metabolic characterization and pathway analysis of berberine protects against prostate cancer

SUPPLEMENTARY MATERIALS

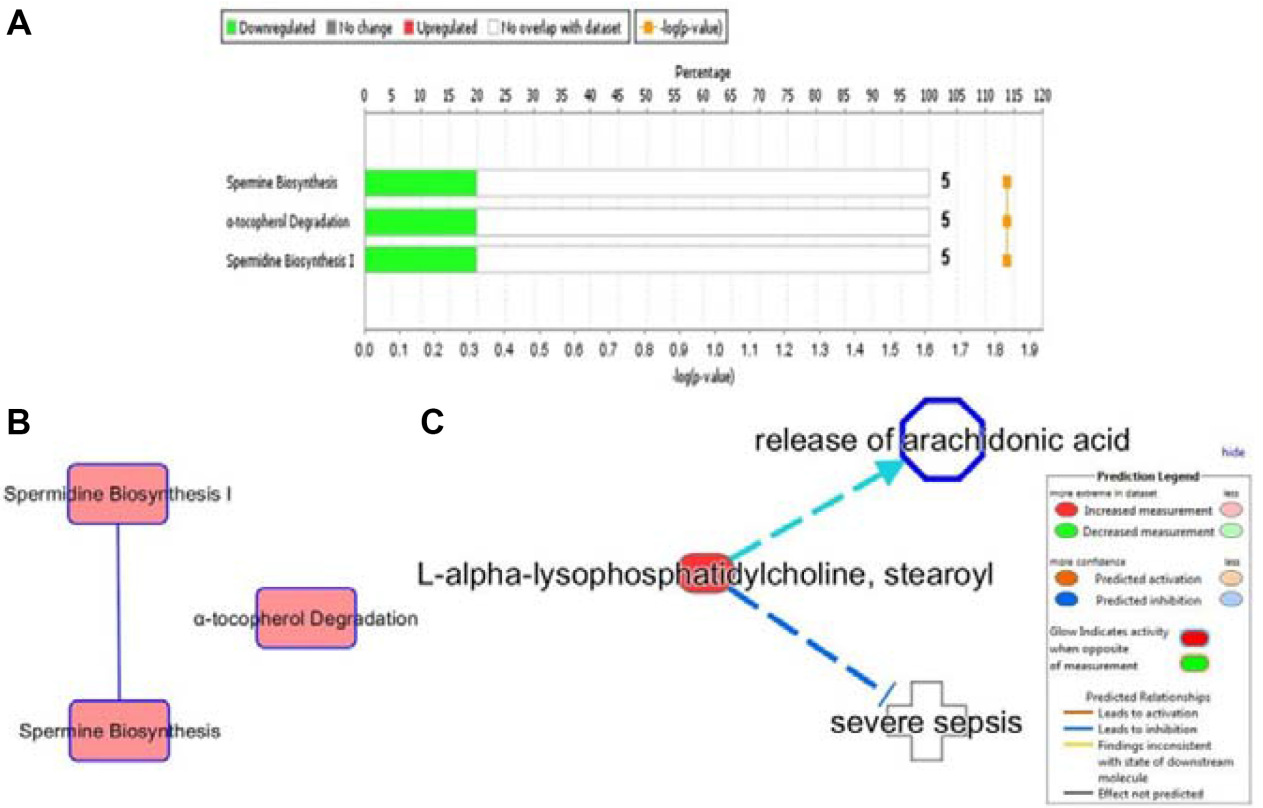

Supplementary Figure 1: Ingenuity pathways analysis of cell metabolite biomarkers. (**A**) Top canonical pathways identified by IPA that are searched of cell metabolite biomarkers; (**B**) IPA analysis reveals a network of signaling pathways searched by cell metabolite biomarkers. (**C**) The biologically active functions network of main cell metabolite biomarkers.

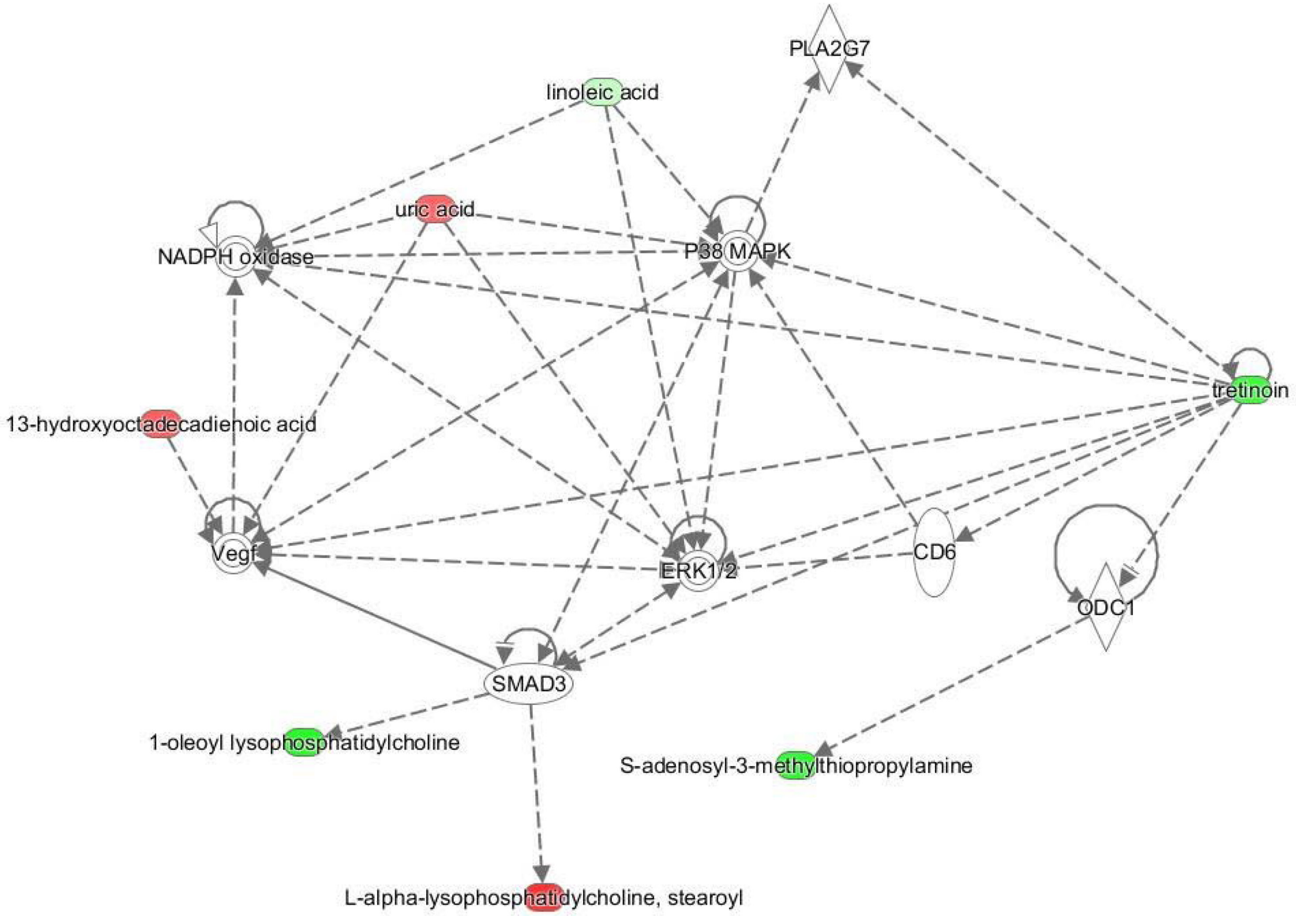

Supplementary Figure 2: The molecular network of berberine against prostate cancer via ingenuity pathways analysis (IPA).

Supplementary Table 1: Cell growth inhibition activity of Berberine treatment (mean ± SD, *n* = 6)

Treatment time (uM)	Proliferation Inhibition Rate (mean ± SD, %)					
	Berberine concentration (μM)					
	1	2.5	5	10	20	50
24 h	0.03 ± 1.35	4.35 ± 0.98	17.67 ± 2.11	36.66 ± 1.53*	37.19 ± 4.54*	55.36 ± 4.23**
48 h	0.07 ± 3.19	18.96 ± 4.90	19.46 ± 1.13	43.71 ± 2.17**	66.45 ± 2.20**	74.76 ± 7.71**
72 h	1.35 ± 0.96	20.55 ± 0.99*	28.08 ± 1.37*	55.78 ± 2.80**	77.53 ± 3.76**	88.80 ± 4.38**

*$P < 0.05$, **$P < 0.01$ vs cell control group.

Supplementary Table 2: The result of relative tumor volume (RTV), tumor relative value-added rate (TRAR) and tumor inhibition rate (TIR) in model group and treatment group ($\overline{x} \pm S$, $n = 5$)

Time (d)	Model group		Treatment group			
	Tumors weight(mg)	RTV (mm^3)	Tumors weight(mg)	RTV (mm^3)	TRAR (%)	TIR (%)
7		4.9 ± 1.9		2.0 ± 0.7	42.1*	——
14		9.9 ± 4.6		3.1 ± 1.0	30.7*	——
21		16.8 ± 5.5		6.0 ± 2.0	35.9**	——
28	2986.8 ± 459.7	27.8 ± 6.3	1753.2 ± 279.5	9.0 ± 2.4	32.4**	41.1**

*P < 0.05, **P < 0.01 vs model group.

Supplementary Table 3: The IOD value of PSA, AR, COX-2, Bcl-2 and Caspase-3 in model group and treatment group (x ± S, $n = 5$)

Group	PSA	AR	COX-2	Bcl-2	Caspase-3
Model group	0.32 ±0.09	0.32 ± 0.08	0.32 ± 0.07	0.30 ± 0.03	0.18 ± 0.07
Treatment group	0.25 ± 0.06*	0.24 ± 0.03*	0.22 ± 0.09*	0.24 ± 0.05*	0.27 ± 0.06*

*P < 0.05 vs model group

Supplementary Table 4: Serum biomarker candidates in prostate cancer model nude mouse identified by UPLC-Q/TOF-MS/MS

No.	Rt (min)	m/z determined	m/z calculated	Ion form	Error (ppm)	Formular	Metabolite Name	Trend	Metabolomic pathway	HMDB
1	0.78	191.0414	191.0439	$[M+H]^+$	-1.0	$C_6H_{14}N_4O_3$	N-(ω)-Hydroxyarginine	↓	Arginine and proline metabolism	HMDB04224
2	0.89	169.0366	169.0362	$[M+H]^+$	2.4	$C_5H_4N_4O_3$	Uric acid	↑	Purine metabolism	HMDB00289
3	0.89	191.0193	191.0192	$[M-H]^-$	0.5	$C_6H_8O_7$	Isocitric acid	↑	Citrate cycle	HMDB00193
4	1.58	165.0558	165.0552	$[M+H]^+$	3.6	$C_9H_8O_3$	2-Hydroxycinnamic acid	↓	Biosynthesis of secondary metabolites	HMDB02641
5	4.57	415.1960	415.1968	$[M-H]^-$	-1.9	$C_{19}H_{30}O_5S$	Androsterone sulfate	↑	Steroid hormone biosynthesis	HMDB02641
6	4.63	329.2122	329.2090	$[M-H]^-$	3.2	$C_{18}H_{34}O_5$	9,12,13-TriHOME	↓	Linoleic acid metabolism	HMDB04708
7	4.67	333.2067	333.2066	$[M-H]^-$	0.3	$C_{20}H_{30}O_4$	Prostaglandin A2	↓	Arachidonic acid metabolism	HMDB02752
8	5.24	335.2226	335.2222	$[M-H]^-$	3.0	$C_{20}H_{32}O_4$	Prostaglandin A1	↓	Arachidonic acid metabolism	HMDB02656
9	5.64	301.2173	333.2168	$[M+H]^+$	1.7	$C_{20}H_{28}O_2$	All-trans-retinoic acid	↓	Retinol metabolism	HMDB01852
10	5.84	378.2405	378.2409	$[M-H]^-$	-0.4	$C_{18}H_{38}NO_5P$	Sphingosine 1-phosphate	↓	Sphingolipid metabolism	HMDB00277
11	5.84	518.3253	518.3243	$[M+H]^+$	1.9	$C_{26}H_{48}NO_7P$	LysoPC(18:3(6Z,9Z,12Z))	↑	Glycerophospholipid metabolism	HMDB10387
12	6.15	295.2232	295.2273	$[M-H]^-$	-4.1	$C_{18}H_{32}O_3$	13S-hydroxyoctadecadienoic acid	↑	Linoleic acid metabolism	HMDB04667
13	6.15	880.5165	880.5151	$[M-H]^-$	2.7	$C_{52}H_{84}NO_8P$	PC(22:6(4Z,7Z,10Z,13Z,16Z,19Z)/22:4(7Z,10Z,13Z,16Z))	↓	Choline metabolism in cancer	HMDB08745
14	6.57	313.1804	313.1804	$[M-H]^-$	0.0	$C_{20}H_{26}O_3$	4-oxo-Retinoic acid	↓	Retinol metabolism	HMDB06285
15	6.67	564.5334	564.5315	$[M-H]^-$	3.4	$C_{36}H_{71}NO_3$	Ceramide (d18:1/18:0)	↑	Sphingolipid metabolism	HMDB04950
16	6.69	398.3368	398.3316	$[M+H]^+$	-4.8	$C_{22}H_{39}NO_5$	PGF2a ethanolamide	↑	Sphingolipid metabolism	HMDB13628
17	7.02	184.0739	184.0738	$[M+Na]^+$	0.1	$C_{10}H_{11}NO$	Tryptophanol	↑	Tryptophan metabolism	HMDB03447
18	7.27	319.2263	319.2273	$[M-H]^-$	-3.1	$C_{20}H_{32}O_3$	16(R)-HETE	↑	Arachidonic acid metabolism	HMDB04680
19	7.31	303.2323	303.2324	$[M+H]^+$	-0.3	$C_{20}H_{30}O_2$	Eicosapentaenoic acid	↓	Biosynthesis of unsaturated fatty acids	HMDB01999
20	7.13	482.3615	482.3612	$[M+H]^+$	0.6	$C_{30}H_{59}NO_3$	Ceramide (d18:1/12:0)	↓	Sphingolipid metabolism	HMDB04947
21	7.45	508.3780	508.3764	$[M+H]^+$	3.1	$C_{26}H_{54}NO_6P$	LysoPC(P-18:0)	↓	Glycerophospholipid metabolism	HMDB13122
22	8.01	568.3633	568.3625	$[M-H]^-$	1.4	$C_{30}H_{52}NO_7P$	LysoPC(22:5(4Z,7Z,10Z,13Z,16Z))	↑	Glycerophospholipid metabolism	HMDB10402
23	8.32	482.3265	482.3247	$[M+H]^+$	4.6	$C_{23}H_{48}NO_7P$	LysoPC(15:0)	↓	Glycerophospholipid metabolism	HMDB10381
24	8.46	506.3615	506.3610	$[M+H]^+$	0.8	$C_{26}H_{52}NO_6P$	LysoPC(P-18:1(9Z))	↑	Glycerophospholipid metabolism	HMDB10408
25	10.24	303.2324	303.2324	$[M-H]^-$	0.0	$C_{20}H_{32}O_2$	Arachidonic acid	↑	Arachidonic acid metabolism	HMDB01043
26	10.43	376.3201	376.3216	$[M+H]^+$	-4.0	$C_{24}H_{41}NO_2$	Adrenoyl ethanolamide	↓	Sphingolipid metabolism	HMDB13626
27	10.78	279.2331	279.2324	$[M-H]^-$	2.5	$C_{18}H_{32}O_2$	Linoleic acid	↑	Linoleic acid metabolism	HMDB00673
28	10.88	813.6826	813.6751	$[M+H]^+$	3.5	$C_{47}H_{93}N_2O_6P$	SM(d18:1/24:1(15Z))	↑	Sphingolipid metabolism	HMDB12107
29	10.93	303.3010	303.3028	$[M+Na]^+$	0.5	$C_{20}H_{40}Na$	8-Isoprostane	↓	Arachidonic acid metabolism	HMDB04659
30	11.48	319.2963	319.2977	$[M+Na]^+$	1.8	$C_{20}H_{40}O$	Thromboxane	↓	Arachidonic acid metabolism	HMDB03208

Supplementary Table 5: Results of metabolism pathways analysis based on KEGG database

No.	Pathway Name	Total	Expected	Hits	-log(p)	Holm p	FDR	Impact
1	Linoleic acid metabolism	6	0.10162	3	9.4	8.27E-05	0.0069	1
2	Arachidonic acid metabolism	36	0.60974	4	5.9531	0.002598	0.1065	0.32601
3	Sphingolipid metabolism	21	0.35568	3	5.3705	0.004652	0.1272	0.31078
4	Biosynthesis of unsaturated fatty acids	42	0.71136	3	3.4487	0.031785	0.6516	0
5	Glycerophospholipid metabolism	30	0.50812	2	2.4115	0.089685	1	0.18333
6	alpha-Linolenic acid metabolism	9	0.15243	1	1.9457	0.14288	1	0
7	Retinol metabolism	16	0.271	1	1.426	0.24027	1	0.22754
8	Glyoxylate and dicarboxylate metabolism	18	0.30487	1	1.324	0.26608	1	0
9	Citrate cycle (TCA cycle)	20	0.33874	1	1.2342	0.29105	1	0.04132
10	Arginine and proline metabolism	44	0.74524	1	0.62754	0.5339	1	0.01198
11	Purine metabolism	68	1.1517	1	0.36263	0.69584	1	0.02077

Note: Total: the total number of the compound in metabolism pathways; Hits: the number of exact matches in the uploaded data; Raw P: the original *P* value through the pathway analysis. Impact: the pathway impact value through topological analysis.

Supplementary Table 6: Cell biomarker candidates in prostate cancer cell model identified by UPLC-Q/TOF-MS/MS

No.	Rt (min)	m/z determined	m/z calculated	Ion form	Error (ppm)	Formular	Metabolite Name	Trend	HMDB
1	6.57	524.3748	524.3716	[M+H]+	0.4	$C_{26}H_{54}NO_7P$	LysoPC(18:0)	↑	HMDB10384
2	5.65	522.3590	522.3560	[M+H]+	3.0	$C_{26}H_{52}NO_7P$	LysoPC(18:1(9Z))	↓	HMDB02815
3	5.62	496.3435	406.3457	[M+H]+	−4.4	$C_{29}H_{47}NO_4$	Clupanodonyl carnitine	↓	HMDB06496
4	4.45	415.2141	415.2250	[M+H]+	−0.6	$C_{19}H_{37}O_6P$	CPA(16:0/0:0)	↓	HMDB07003
5	5.84	379.0875	379.0875	[M+H]+	0.0	$C_{11}H_{16}N_5O_8P$	7-Methylguanosine 5’-phosphate	↓	HMDB59612
6	0.63	365.1088	365.1084	[M+H]+	1.1	$C_{12}H_{22}O_{11}$	Galactinol	↓	HMDB05826
7	5.81	351.0919	351.0917	[M+H]+	0.3	$C_{12}H_{16}N_4O_7$	7-Hydroxy-6-methyl-8-ribityl lumazine	↓	HMDB04256
8	6.04	349.2379	349.3470	[M+H]+	2.6	$C_{21}H_{31}O_4$	3b,15b,17a-Trihydroxy-pregnenone	↓	HMDB00353
9	5.85	317.1186	317.1137	[M+H]+	2.5	$C_{14}H_{18}N_2O_5$	Glutamylphenylalanine	↓	HMDB00594
10	5.85	301.1416	301.1445	[M+H]+	-2.9	$C_{16}H_{22}O_4$	Alpha-CEHC	↓	HMDB01518
11	0.56	299.1135	299.1131	[M+H]+	1.3	$C_{14}H_{18}O_7$	2-Phenylethanl glucuronide	↓	HMDB10350
12	3.17	157.0513	157.0501	[M+H]+	1.2	$C_7H_8O_4$	2,3-Methyleneglutaric acid	↓	HMDB59731
13	5.81	459.1158	459.1139	[M+H]+	4.1	$C_{17}H_{23}N_4O_9P$	FMNH2	↓	HMDB01142
14	7.90	353.1870	353.1865	[M+H]+	1.4	$C_{14}H_{23}N_6O_3S$	S-Adenosylmethioninamine	↓	HMDB00988
